# Cortical morphometric inverse divergence in attention-deficit/hyperactivity disorder correlates with cell-type-specific, laminar-specific and developmental transcriptomic signatures

**DOI:** 10.1017/S003329172610405X

**Published:** 2026-05-11

**Authors:** Yexian Zeng, Li Yang, Zaixu Cui, Qingjiu Cao

**Affiliations:** 1 Peking University Sixth Hospital, Beijing, China; 2 Peking University Institute of Mental Health, Beijing, China; 3 NHC Key Laboratory of Mental Health, Peking University, Beijing, China; 4 National Clinical Research Center for Mental Disorders, Peking University Sixth Hospital, Beijing, China; 5 Beijing Key Laboratory for Big Data Innovative Application of Child and Adolescent Mental Disorders, Beijing, China; 6 Beijing Institute for Brain Research, Chinese Academy of Medical Sciences & Peking Union Medical College, Beijing, 102206, China; 7 Chinese Institute for Brain Research, Beijing, 102206, China

**Keywords:** ADHD combined subtype, cell-type-specific signatures, cortical gene expression, cortical layers enrichment developmental stages assessment, morphometric inverse divergence network, transcriptional patterns

## Abstract

**Background:**

Attention-deficit/hyperactivity disorder (ADHD) is a neurodevelopmental condition with widespread brain structure alterations. However, the relationship between macroscale cortical organization and microscale molecular mechanisms remains unclear, particularly regarding the neurobiological mechanisms shared between the full ADHD cohort and its combined subtype (ADHD-C).

**Methods:**

We analyzed 176 patients with ADHD (105 ADHD-C, 71 ADHD inattentive subtype) and 176 matched typically developing (TD) controls from the ADHD-200 dataset. Morphometric Inverse Divergence (MIND) networks quantified cortical similarity. Partial least squares (PLS) regression linked case–control MIND differences to cortical gene expression, assessing functional enrichment, cell-type specificity, and developmental trajectories.

**Results:**

Neuroanatomically, the ADHD-C subtype exhibited widespread increases in regional MIND values, particularly in temporal and parietal cortices, reflecting greater inter-regional morphological homogeneity. PLS regression revealed that these MIND alterations were spatially correlated with a specific transcriptomic signature (PLS1+). These PLS1+ genes were significantly enriched in mitochondria-related metabolic pathways and showed distinct cortical layer specificity (notably layer V) and developmental stage specificity (from late fetal to late infancy stages). Regarding cell-type specificity, while PLS1+ genes in the full ADHD cohort were significantly enriched in excitatory and inhibitory neurons, the ADHD-C subtype showed similar but trend-level associations. Importantly, the full ADHD cohort and the ADHD-C group shared numerous PLS1-related genes and broad functional pathway enrichment commonalities.

**Conclusions:**

This study links macroscale cortical abnormalities to microscale transcriptional regulation, with pronounced alterations in ADHD-C. The shared genetic and functional profiles between ADHD and its combined subtype underscore common pathological processes, providing novel insights into the neurodevelopmental mechanisms of ADHD.

## Introduction

Attention-deficit/hyperactivity disorder (ADHD) is a common neurodevelopmental condition that usually emerges during childhood (Bochet et al., 2021). It is characterized by persistent patterns of inattention and/or hyperactivity/impulsivity that can significantly limit cognitive function and academic performance (Posner, Polanczyk, & Sonuga-Barke, 2020). In many individuals, these symptoms often persist into adulthood (Cortese et al., 2018; Simon et al., 2009). Recent epidemiological evidence indicates that approximately 5.3% of children and adolescents in China are diagnosed with ADHD (Dong et al., 2025). The Diagnostic and Statistical Manual of Mental Disorders, Fourth Edition (DSM-IV) classifies ADHD into three subtypes: the combined type (ADHD-C), the inattentive type (ADHD-I), and the hyper-impulsive type (ADHD-H) (Guze, 1995).

Although these subtypes are widely used in clinical settings, their identification continues to depend largely on subjective behavioral assessments and rating scales, and there are still no dependable objective biological markers to support diagnosis. Previous neuroimaging research has consistently shown that the variability in clinical symptoms among ADHD subtypes may be linked to differences in brain structure. For instance, patients with the combined subtype (ADHD-C) exhibit significantly reduced gray matter volume in the caudate nucleus and anterior cingulate gyrus compared with those with the inattentive subtype (ADHD-I) (Semrud-Clikeman, Fine, Bledsoe, & Zhu, 2017). In addition, ADHD-I is more commonly associated with abnormalities in the frontoparietal network (Diamond, 2005). However, most traditional neuroimaging studies focus on only one structural measure, such as cortical thickness or gray matter volume (Shaw et al., 2011, 2013; Silk et al., 2016). This narrow focus does not sufficiently capture the complex alterations that occur across the whole-brain network and has contributed to inconsistent findings when comparing ADHD subtypes. Consequently, a more comprehensive and precise approach is needed to characterize the brain’s structural network, allowing for clearer identification of the neuroarchitectural features underlying ADHD and its subtypes.

In recent years, morphological similarity networks (MSNs) have provided a new framework for examining structural coordination in the brain by integrating multiple morphological features, such as volume, thickness, and curvature, to construct a connectome (Seidlitz et al., 2018). However, the MSN approach is limited by its simplified feature representation and its inability to account for variability in feature distribution within cortical regions (Li, Chen, & Lin, 2021; Zhang et al., 2025). To address these limitations, the morphometric inverse divergence (MIND) network was introduced. This method evaluates differences in the distributions of multiple structural features across brain regions using the Jensen–Shannon divergence (Bazinet, Hansen, & Misic, 2023, Sebenius et al., 2023. Although MIND has proven effective in studies of disorders such as major depressive disorder (MDD) (Niu et al., 2026) and early-onset schizophrenia (Xue et al., 2025; Yao et al., 2024), it has not yet been applied to investigate ADHD or its subtypes. Therefore, its potential to elucidate the neural mechanisms underlying subtype-specific heterogeneity remains unexplored.

ADHD also has a strong genetic component. Genome-wide association studies have identified several risk genes that are particularly enriched among those expressed during early brain development (Demontis et al., 2019, 2023). An important unresolved question is whether the macroscale brain network alterations observed in ADHD and its subtypes are associated with these underlying genetic risk factors. Whole-brain transcriptome data from the Allen human brain atlas (AHBA) provide a valuable resource for investigating this relationship (Li et al., 2021; Morgan et al., 2019; Romero-Garcia et al., 2019). Combining MIND-based brain network metrics with spatial gene expression data from the AHBA offers the potential to uncover the genetic basis of ADHD-related brain network abnormalities at a systems level, yielding new insights into the disorder’s pathological mechanisms.

In summary, this study aims to construct MIND networks for ADHD and its subtypes, as well as typically developing (TD) controls, using the multicenter ADHD-200 dataset for the first time. The objectives are to: (1) identify topological network features specific to each subtype, and (2) explore, in combination with AHBA transcriptomic data, the relationship between these abnormal network features and the spatial co-expression patterns of genes associated with ADHD risk. We expect that this study will provide reliable neuroimaging evidence for ADHD and enhance our understanding of its underlying pathological mechanisms by examining the links between brain network alterations and genetic factors.

## Methods

### Participants

We selected data from four sites within the publicly available multisite ADHD-200 dataset: Peking University (PKU), New York University Child Study Center (NYU), Kennedy Krieger Institute (KKI), and Oregon Health & Science University (OHSU). These sites were selected because they provided thorough and consistent clinical assessments, such as complete ADHD rating scales and full-scale intelligence quotient (FSIQ) data, which are crucial for accurate phenotypic characterization and proper group matching. The experimental procedures were approved by the local institutional review board, and written informed consent was obtained from all participants. Individuals in this database were categorized into one of four groups: ADHD-C, ADHD-I, ADHD-H, or TD. To reduce potential confounding factors, we included only children and adolescents diagnosed with ADHD based on validated criteria, such as the DSM-IV, after visually inspecting the image quality. However, the ADHD-H subtype included only six participants, which was too small for reliable statistical analysis. Consequently, this group was excluded from further analysis (Supplementary Method S1).

Propensity score matching was used to control for potential confounding variables during the participant selection process. A logistic regression model was developed with sex and age as covariates to calculate propensity scores, and matching was performed using the caliper method with a caliper value of 0.073. The quality of the matching was assessed using standardized mean differences (SMD). Visual inspection confirmed that the absolute SMDs for sex (<0.001) and age (0.191) were within the acceptable range (<0.2) after matching, demonstrating satisfactory balance between the groups (Stuart, 2010) (Supplementary Figure R1). After screening, a total of 176 ADHD patients and 176 matched TD individuals were included in this study (Supplementary Method S1).

### Imaging preprocessing

T1-weighted images were preprocessed in surface-based space using FreeSurfer v8.0 (https://surfer.nmr.mgh.harvard.edu/fswiki/rel7downloads). The automated recon-all pipeline was used, performing skull-stripping, tissue segmentation, separation of hemispheric and subcortical structures, and the construction of gray/white matter boundaries and pial surfaces. For all included subjects, total intracranial volume (TIV) was calculated for use in subsequent analyses. To control for potential scanner-related variability across the multisite dataset, site effects were adjusted using the nonparametric ComBat approach in MATLAB. The effectiveness of this harmonization procedure in eliminating site effects was confirmed through visual inspection of the data distribution before and after correction (Supplementary Figure R2). Detailed information on image preprocessing is provided in Supplementary Method S2.

### Construction of the MIND network

This study employed the widely used Desikan–Killiany (DK) 308 cortical atlas (Li, Chen, & Lin, 2021; Morgan et al., 2019; Seidlitz et al., 2018), which is based on the standard DK 68-region map (Desikan et al., 2006) and refined through a retrospective algorithm (Romero-Garcia, Atienza, Clemmensen, & Cantero, 2012) to divide the cortex into 308 spatially contiguous regions of approximately equal size, averaging around 5 cm^2^ each. This atlas was selected to balance spatial resolution with anatomical interpretability, consistent with established protocols in recent morphometric similarity research (Chen et al., 2024; Wu et al., 2025; Xue et al., 2025).

The standard atlas was adapted to match the individual cortical anatomy of each participant. At every vertex on the personalized cortical surface, key morphological features were measured, including cortical thickness (CT), gray matter volume (GMV), mean curvature (MC), surface area (SA), and sulcal depth (SD) (Sebenius et al., 2023. To address differences in scale among the morphological features, each feature was normalized across all brain vertices using z-scores, following previous methods (Sebenius et al., 2023. The standardized vertex-level data were then aggregated within the 308 predefined cortical regions to create a multivariate feature distribution for each region. To assess the multidimensional morphological similarity between two cortical regions, we calculated the MIND similarity statistic (Sebenius et al., 2023 based on the transformed Kullback–Leibler (KL) divergence. This measure ranges from 0 to 1, with higher values reflecting greater similarity. Finally, for each participant, a 308 × 308 symmetric matrix was created to represent the MIND network (Supplementary Method S3), with each entry reflecting the pairwise MIND similarity between all 308 cortical regions.

### Comparative analysis of regional MIND indicators

We defined the regional MIND value as the weighted node degree of each cortical region, calculated by averaging the MIND similarity values across all its connections with other regions in the network. To examine the differences in regional MIND values between the ADHD and TD groups, we performed a general linear model (GLM) analysis. In this model, group membership (ADHD versus TD or ADHD subtypes versus TD) was treated as the independent variable, while regional MIND values served as the dependent variable. The analysis controlled for age, sex, TIV, and interactions among these covariates. To understand the observed regional differences within the context of large-scale brain networks, all 308 cortical regions were mapped onto the Yeo-7 functional Network atlas, which is defined based on resting-state functional connectivity (Yeo et al., 2011). For each Yeo network, the mean MIND value was calculated across all constituent regions. The same GLM model, including the same covariates, was used to evaluate differences in mean MIND values between the ADHD and TD groups at the Yeo network level. All statistical analyses, performed at the regional and Yeo network levels, applied Bonferroni correction for multiple comparisons (*p*_Bonf_ < 0.05) to rigorously control the family-wise error rate (FWER) across the defined anatomical regions.

Within the ADHD group, we further examined the relationship between regional MIND values showing significant group differences and clinical symptoms, including inattention and hyperactivity/impulsivity, using partial correlation analysis. The resulting correlations were corrected for multiple comparisons using the false discovery rate (FDR) method to balance statistical power with false-positive control in these exploratory clinical associations.

### Transcriptomic profiling and PLS regression analysis in ADHD

Gene expression data were sourced from six postmortem brains in the AHBA, covering 3,702 spatially distinct tissue samples (http://human.brain-map.org) (Hawrylycz et al., 2012). Because only two donors provided right-hemisphere data, the analysis was restricted to the left hemisphere, which was divided into 152 regions according to the DK-308 atlas. Gene expression data were preprocessed and mapped to these regions using the abagen toolbox (https://github.com/rmarkello/abagen), resulting in a final expression matrix of 152 regions by 15632 genes.

We applied partial least squares (PLS) regression to examine the association between regional gene expression patterns and case–control differences in MIND among individuals with ADHD (Abdi & Williams, 2013). In this model, gene expression levels across all genes served as predictors, while regional MIND *t*-statistics were used as the response variable. PLS extracts latent components that capture the maximal covariance between genetic and neuroanatomical measures. The significance of each component was evaluated using spin-based permutation testing with 10,000 iterations (Vasa et al., 2018), and only components explaining more than 20% of the variance with *p* < 0.05 were retained (Abdi & Williams, 2013).

Gene-wise stability was assessed using bootstrap resampling with 10,000 iterations. *Z*-scores were calculated by dividing the weights by their bootstrap standard error, and genes with *p*_FDR_ < 0.001 were considered significant. FDR correction was selected for this high-dimensional transcriptomic dataset to minimize Type II errors (false negatives) while maintaining a controlled proportion of false discoveries.

### Assigning ADHD-related genes to cortical layers, developmental stages, and cell types

For the developmental trajectory analysis, we used cell-type-specific expression analysis (CSEA) (http://doughertytools.wustl.edu/CSEAtool.html). Enrichment scores for each gene set were computed across 15 developmental stages, from early fetal to young adulthood, and across 12 brain regions. We further investigated the relationship between PLS-weighted genes and developmental time in ADHD using developmental gene expression richness analysis. This approach highlighted the spatiotemporal expression patterns of genes associated with ADHD pathology across different brain regions.

To further confirm the cell-type specificity associated with MIND regional alterations, we compared the PLS1+/− ranked genes with gene sets representing seven cortical cell types: glial cells, oligodendrocytes, endothelial cells, astrocytes, oligodendrocyte precursor cells (OPCs), excitatory neurons (Neuno-Ex), and inhibitory neurons (Neuno-In) (Li, Chen, & Lin, 2021). These cell-type markers were obtained from five independent single-cell studies of human postmortem cortical tissue (Seidlitz et al., 2020) (Supplementary Method S4).

### Null model

To control for potential confounding effects of spatial autocorrelation, we applied a spatial permutation method using the spin test procedure (Vasa et al., 2018). This approach maintains the spatial organization of cortical data by randomly rotating spherical projections of the brain maps, producing null distributions of Spearman correlation coefficients. For each analysis, 10,000 permutations were performed to generate a robust null model. The *p*_spin_ value was calculated as the proportion of null correlations that exceed the observed correlation.

### Validation of reproducibility

**Figure 1.**
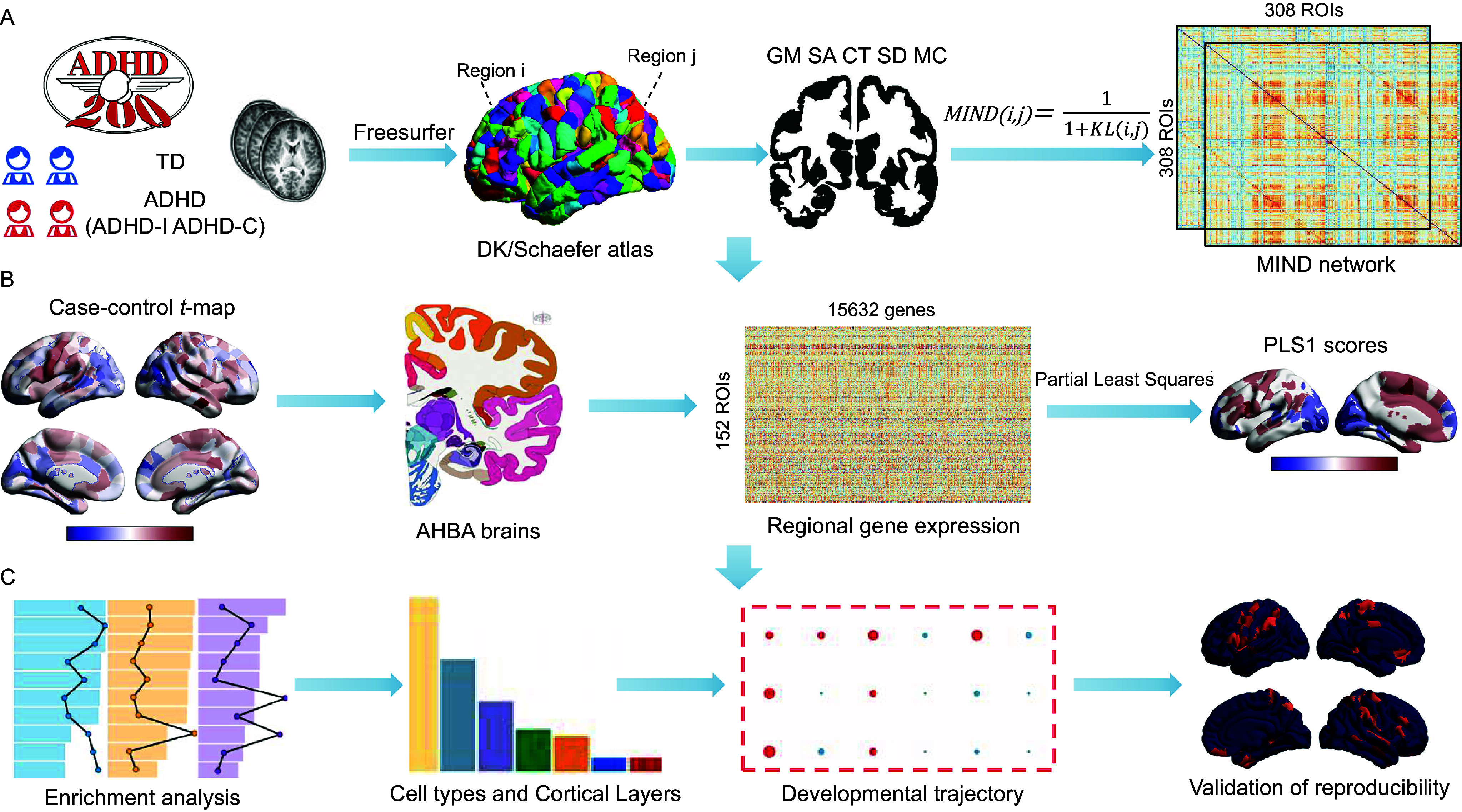
Overview of the analytical workflow. (A) MIND network construction. A 308 × 308 regional MIND matrix was constructed using five cortical features: gray matter volume, surface area, sulcal depth, mean curvature, and cortical thickness. Regional MIND values were computed by averaging all connections per region without thresholding. (B) Gene expression data were obtained from the AHBA and mapped to the left cortical regions, forming a regional expression matrix. (C) Transcriptomic association and functional annotation. PLS regression was applied to identify associations between regional gene expression and MIND alterations. Downstream analyses included genetic correlation with psychiatric disorders, functional enrichment of PLS-weighted genes, and developmental, laminar, and cell-type-specific expression profiling. *Note:* MIND, morphometric inverse divergence; AHBA, Allen human brain atlas; PLS, partial least squares.

To confirm the reliability and robustness of our results, we conducted several validation analyses: (1) examining the stability of case–control MIND *t*-maps without adjusting for TIV; (2) assessing the consistency of MIND differences between TD and ADHD subtypes (ADHD-C and ADHD-I) while controlling for age, sex, FSIQ, inattentive scores, hyperactive/impulsive scores, TIV, and all two-way interactions among these variables; (3) evaluating the impact of threshold selection on MIND results by computing network metrics across a range of connection densities (10%–90% in 20% increments) based on MIND differences between healthy controls and ADHD cases (Li, Chen, & Lin, 2021); (4) assessing the reproducibility of our core findings in a large, independent external validation cohort (*N*_total_ = 277). This independent dataset was aggregated from four sources: the NeuroIMAGE site from ADHD-200 (not included in the discovery set) and three OpenNeuro datasets (ds002424, ds005899, and ds004605), comprising 127 ADHD patients and 150 TD controls; (5) repeating the network diffusion analysis using the Schaefer400 atlas to verify the reliability of the results; (6) validating functional enrichment findings through a meta-analysis of multiple gene lists, including genes linked to ADHD in genome-wide association studies (GWAS). The workflow and technical details for this study are illustrated in Figure 1.

## Results

### Demographic and clinical characteristics

Table 1 indicates that children with ADHD and the TD group did not differ significantly in age (*p* = 0.074) and sex (*p* = 1). However, the ADHD group exhibited significantly higher inattention (*p* < 0.001) and hyperactivity/impulsivity (*p* < 0.001) scores, as well as significantly lower FSIQ scores (*p* < 0.001) compared with the TD group. When comparing ADHD subtypes with the TD group, significant differences were found in inattention symptoms (F_(2,349)_ = 93.25, *p* < 0.001, with scores following the pattern ADHD-C > ADHD-I > TD, and in hyperactivity/impulsivity behavior (F_(2,349)_ = 106.59, *p* < 0.001), also following ADHD-C > ADHD-I > TD. Furthermore, the TD group and individuals with ADHD-I were significantly older than those with ADHD-C (F_(2,349)_ = 8.1345, *p* < 0.001). In addition, FSIQ scores were significantly higher in the TD than in both ADHD subtypes (F_(2,349)_ = 20.09, *p* < 0.001). The chi-square test indicated a statistically significant difference in sex distribution among the three groups (χ^2^_(2)_ = 7.70, *p* = 0.021), with post hoc analysis showing that this difference was significant only between ADHD-C and ADHD-I (*p* = 0.017).

**Table 1.** Demographic and clinical characteristics

	TD (N = 176)	ADHD (N = 176)	*P* value *TD versus ADHD*	ADHD subtypes		χ^2^/ *F Value* ADHD-C *versus* ADHD-I *versus* TD	*P Value* ADHD-C *versus* ADHD-I *versus* TD	Follow-up tests
				ADHD-C (N = 105)	ADHD-I (N = 71)			
Male^a^	120 (68.2%)	120 (68.1%)	1	80 (76.1%)	40 (56.3%)	7.70^a^	**0.021** ^a^	**ADHD-C > ADHD-I**
Age (years)	10.73 ± 2.47	10.27 ± 2.36	0.074	9.82 ± 2.12	10.95 ± 2.53	8.14	**< 0.001**	**TD, ADHD-I > ADHD-C**
Inattentive	39.25 ± 13.63	62.49 ± 20.57	**< 0.001**	67.18 ± 17.37	55.54 ± 22.97	93.25	**< 0.001**	**ADHD-C > ADHD-I > TD**
Hyper/impulsive	38.97 ± 14.45	59.15 ± 22.85	**< 0.001**	68.28 ± 19.00	45.65 ± 21.44	106.59	**< 0.001**	**ADHD-C > ADHD-I > TD**
FSIQ	114.74 ± 13.45	106.19 ± 13.40	**< 0.001**	107.66 ± 13.56	104.01 ± 12.94	20.09	**< 0.001**	**TD > ADHD-C, ADHD-I**

*Note:* Bolded values are *p* < 0.05; the results show as mean ± SD, SD: standard deviation; ADHD, Attention-Deficit/Hyperactivity Disorder (the full ADHD cohort); ADHD-C, ADHD of Combined type; ADHD-I, ADHD of Inattention type; TD, Typically Developing controls; FSIQ: Full-scale intelligence quotient.

a Pearson chi-square test.

### ADHD-related changes in MIND

**Figure 2.**
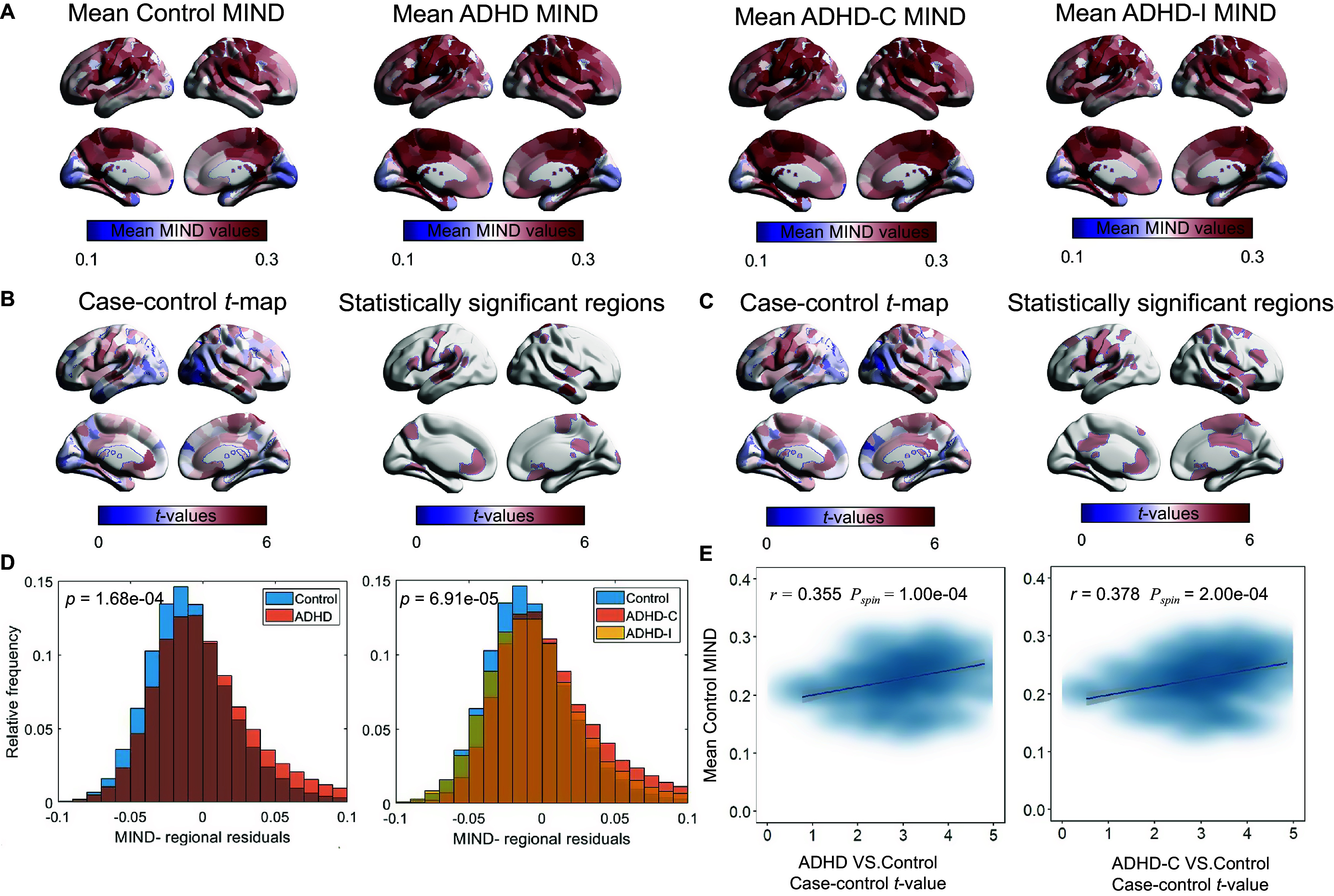
Group differences and cognitive correlates of morphometric similarity in ADHD. (A) Regional MIND distributions across the full ADHD cohort, ADHD-C and ADHD-I subtypes, and TD controls. Patient groups showed regional patterns of MIND similar to TD. (B) Statistical maps of regional MIND differences between the full ADHD cohort and TD controls. The *t*-values >0 means ADHD-C > TD (left: unthresholded; right: *p*_Bonf_ < 0.05). (C) Regional MIND differences between ADHD-C and TD controls, with the same color scheme and thresholding as in (B). (D) Frequency distributions of regional MIND differences between ADHD and TD (left), and between ADHD-C and TD (right), after controlling for age, sex, and TIV. (E) Scatter plots showing spatial correlation between mean MIND in TD controls and case–control *t*-values across 308 regions. Left: ADHD versus TD, *r*_(308)_ = 0.355, *p*_spin_ = 1.0 × 10^−4^; Right: ADHD-C versus TD, *r*_(308)_ = 0.378, *p*_spin_ = 2.0 × 10^−4^. *Note:* ADHD, attention deficit/hyperactivity disorder; ADHD-C, attention deficit/hyperactivity disorder combined type; TD, typically developing; MIND, Morphometric Inverse Divergence; *p*_Bonf,_ *p*-values after Bonferroni correction; *p*_spin_, spin test.

Regional MIND is defined as the average weighted degree of nodes within the MIND network. Although the overall distribution of regional MIND was similar between the ADHD and TD groups, with higher values in the temporal and parietal cortices and lower values in the occipital cortex (Figure 2A), analysis identified significant differences between the groups in 27 cortical regions (*p*_Bonf_ < 0.05). The brain regions showing the most pronounced differences between groups included the right middle temporal gyrus (part 3; *p*_Bonf_ = 5.4 × 10^−4^), left superior temporal gyrus (part 5, *p*_Bonf_ = 1.1 × 10^−3^), and left rostral anterior cingulate (part 1, *p*_Bonf_ = 8.05× 10^−3^) (Figure 2B; Supplementary Table R1). Furthermore, global MIND was defined as the average of regional MIND values across all cortical regions. MIND analysis revealed that the overall cortical morphological profile of the ADHD group differed significantly from that of the TD group (*p*_Bonf_ = 1.68 × 10^−4^) (Figure 2D).

In the subtype analysis, only the ADHD-C subtype exhibited a widespread increase in MIND values across 54 cortical regions compared with the TD group (Figure 2A,C). The largest effect was observed in the right middle temporal gyrus (part 3; *t* = 5.617, *p*_Bonf_ = 1.24 × 10^−5^), while the most notable abnormalities were detected in the left precentral gyrus (part 4; *t* = 4.866, *p*_Bonf_ = 5.35 × 10^−4^), right superior parietal (part 2; *t* = 4.866, *p*_Bonf_ = 5.36 × 10–4), and right anterior insula (*t* = 3.981, *p*_Bonf_ = 0.026) and several other cortical areas (Supplementary Table R2). Similarly, global MIND values showed significant differences between the ADHD-C group and the TD group (*p*_Bonf_ = 6.91 × 10^−5^) (Figure 2D). A significant positive correlation was found between mean regional MIND in controls and the case–control *t*-map for both the full ADHD cohort (*r*_(308)_ = 0.355, *p*_spin_ = 1 × 10^−4^) and the ADHD-C subtype (*r*_(308)_ = 0.378, *p*_spin_ = 2 × 10^−4^) (Figure 2E). These findings indicate that regions with higher baseline connectivity tend to exhibit larger case–control differences in MIND.

**Figure 3.**
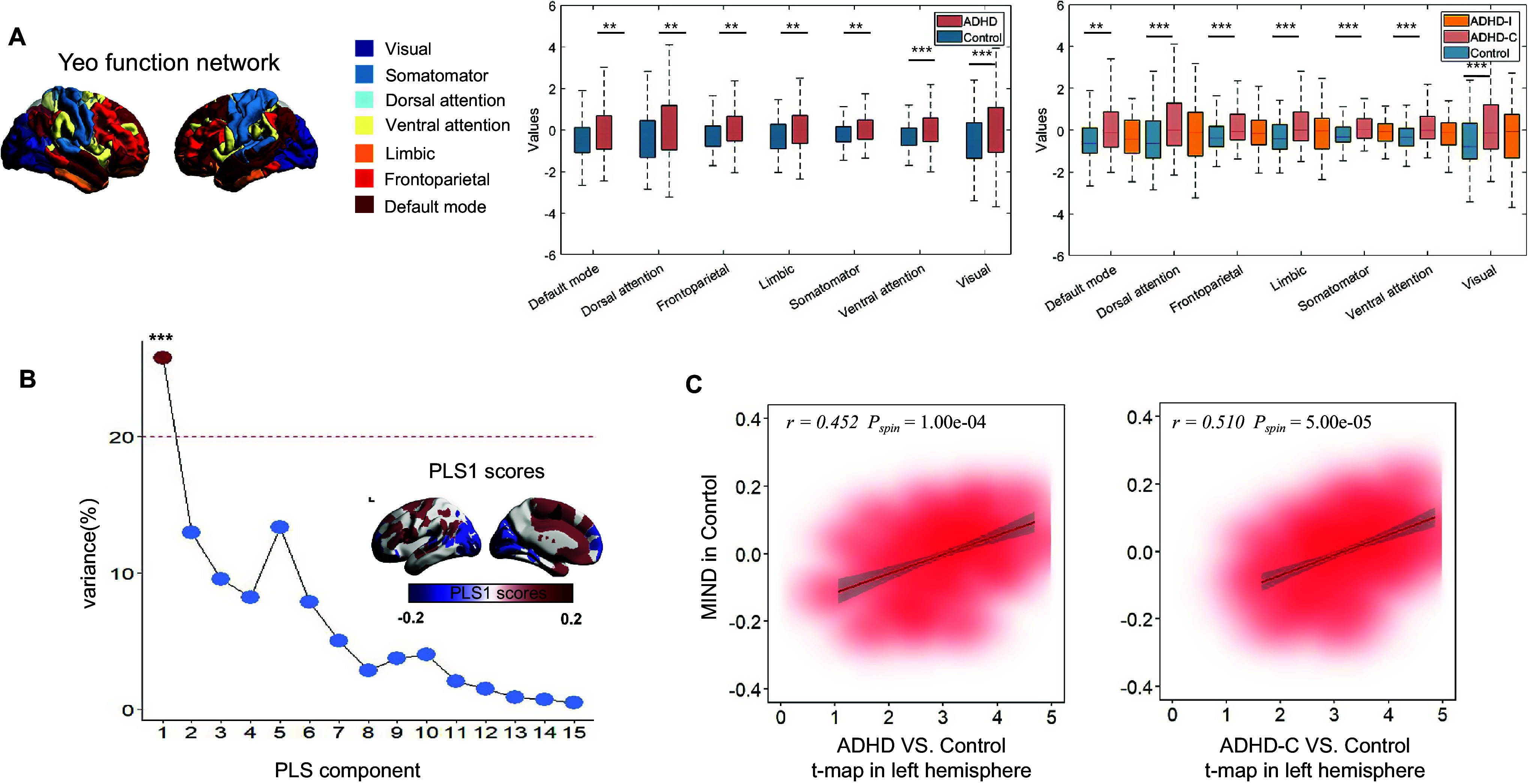
Altered Cortical MIND Patterns and Neurogenetic Correlates in ADHD and ADHD-C. (A) (left) Yeo–7 functional networks atlas are labeled according to the color scheme of their associated functional networks; (Middle) ADHD patients showed significantly increased MIND across all functional networks compared to TD controls. (B) Variance in case–control MIND differences explained by the top 15 PLS components. Only the first component (PLS1) accounted for more than 20% of the variance (26.94%) and was statistically significant after controlling for spatial autocorrelation (*p*_spin_ < 0.05). *** *p* < 0.001. (C) PLS1 component scores exhibited a significant positive spatial correlation with case–control MIND *t*-statistics in both the full ADHD cohort (left; *r* = 0.510, *p*_spin_ = 5 × 10^−5^, gray band indicates 95% CI) and the ADHD-C subgroup (right; *r* = 0.510, *p*_spin_ = 5 × 10^−5^, gray band indicates 95% CI). ADHD, attention-deficit/hyperactivity disorder; ADHD-C, attention-deficit/hyperactivity disorder combined type; TD, typically developing; MIND, morphometric inverse divergence; PLS, partial least squares; *p*_spin_, spin test.

We also investigated abnormal MIND patterns within the functional Yeo 7 networks in the ADHD group. The analysis revealed that the full ADHD cohort exhibited significantly higher MIND values across all seven Yeo resting-state networks. This network-level abnormality was only observed when comparing the ADHD-C subtype with the TD group (Figure 3A; Supplementary Table R3). Although we examined the potential relationship between MIND abnormalities and clinical measures, including inattention, hyperactivity/impulsivity, and FSIQ, using Spearman’s correlation while controlling for relevant variables, no significant associations were detected (Supplementary Table R4).

### Transcriptional patterns associated with regional changes in MIND

To investigate the molecular mechanisms underlying abnormal MIND alterations, we analyzed genome-wide expression profiles of 152 brain regions (15,631 genes) to assess their spatial association with case–control MIND differences using PLS. In the full ADHD cohort (explained variance = 20.32%, *p*_spin_ = 0.005; Supplementary Figure R3) and the ADHD-C subgroup (explained variance = 26.94%, *p*_spin_ < 0.001, Figure (Figure 3B), only the first component (PLS1) was statistically significant. In the ADHD-C group, the spatial loadings of PLS1 showed a positive correlation with the case–control *t*-map (*r* = 0.452, *p*_spin_ = 1.0 × 10^−4^), indicating that PLS1-weighted genes are preferentially overexpressed in brain regions with increased MIND. The ADHD-C subtype exhibited a stronger spatial association (*r* = 0.510, *p*_spin_ = 5.10 × 10^−4^), with PLS1 accounting for variance well above chance levels (Figure 3C **)**.

**Figure 4.**
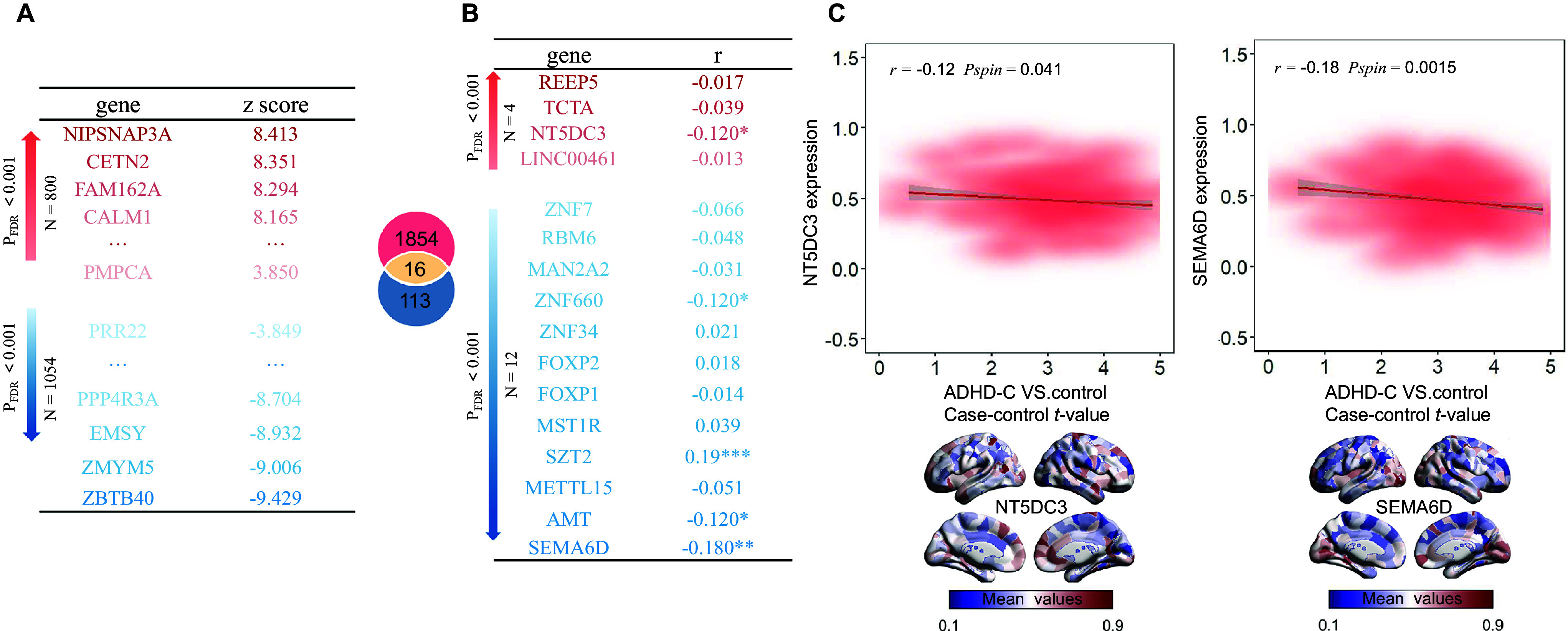
Gene expression profiles related to case–control *t*-values in ADHD-C. (A) Ranked weights of genes contributing to PLS1. (B) Correlation between ADHD risk genes derived from genome-wide association studies and case–control *t*-values, revealing one significant positive and four significant negative associations. * *p* < 0.05, *** *p* < 0.001. Genes labeled in red denote PLS1+ genes; those in blue indicate PLS1− genes. (C) (left) Association between *NT5DC3* (PLS1+) expression and case–control *t*-values (*r* = −0.12, *p* = 0.041), with left-hemisphere expression map of *NT5DC3.* (right) Association between *SEMA6D* (PLS1−) expression and case–control *t*-values (*r* = −0.18, *p* = 0.001), with left-hemisphere expression map of *SEMA6D. Note:* ADHD, attention-deficit/hyperactivity disorder; ADHD-C, attention-deficit/hyperactivity disorder combined type; TD, typically developing; PLS, partial least squares.

Since ADHD-C exhibited the most pronounced morphological and functional network abnormalities in the previous analysis, including elevated MIND in 54 cortical regions and widespread alterations across the Yeo network, subsequent molecular investigations were concentrated on the ADHD-C subtype. Following previous studies (Li, Chen, & Lin, 2021; Xue et al., 2023), genes in PLS1 were ranked according to their *z*-scores (Figure 4A). In the ADHD-C group, 1,854 genes were significantly associated with PLS1 (*p*_FDR_ < 0.001), of which 800 exhibited positive normalized weights (PLS1+) and 1,054 exhibited negative normalized weights (PLS1−). Next, to determine whether the PLS1+/− genes were enriched for ADHD risk genes, we compared them with those identified in the latest large-scale GWAS (Demontis et al., 2023). This analysis revealed 16 overlapping genes out of 129 reported ADHD risk genes (*p*_FDR_ < 0.05; Figure 4B). Among these genes, only one displayed a significant positive correlation with the case–control *t*-map, whereas four genes showed significant negative correlations (*p*_FDR_ < 0.05; Figure 4B). The only correlated PLS1+ gene was *NT5DC3* (*r* = −0.12, *p* = 0.041), and the correlated PLS1− gene (|z|_max_) was *SEMA6D* (*r* = −0.18, *p* = 0.002) (Figure 4C).

### Functional annotation of PLS-weighted genes related to regional changes in MIND

To further explore the functional significance of MIND-associated gene alterations, PLS1+ and PLS1− genes were analyzed using Metascape for enrichment in gene ontology (GO) biological processes, Kyoto Encyclopedia of Genes and Genomes (KEGG) pathways, and gene sets linked to six other brain disorders. Neither PLS1+ nor PLS1− genes demonstrated significant associations with dysregulated genes related to other psychiatric disorders in either the full ADHD cohort or ADHD-C (*p*_FDR_perm_ > 0.05) (Supplementary Figure R4).

**Figure 5.**
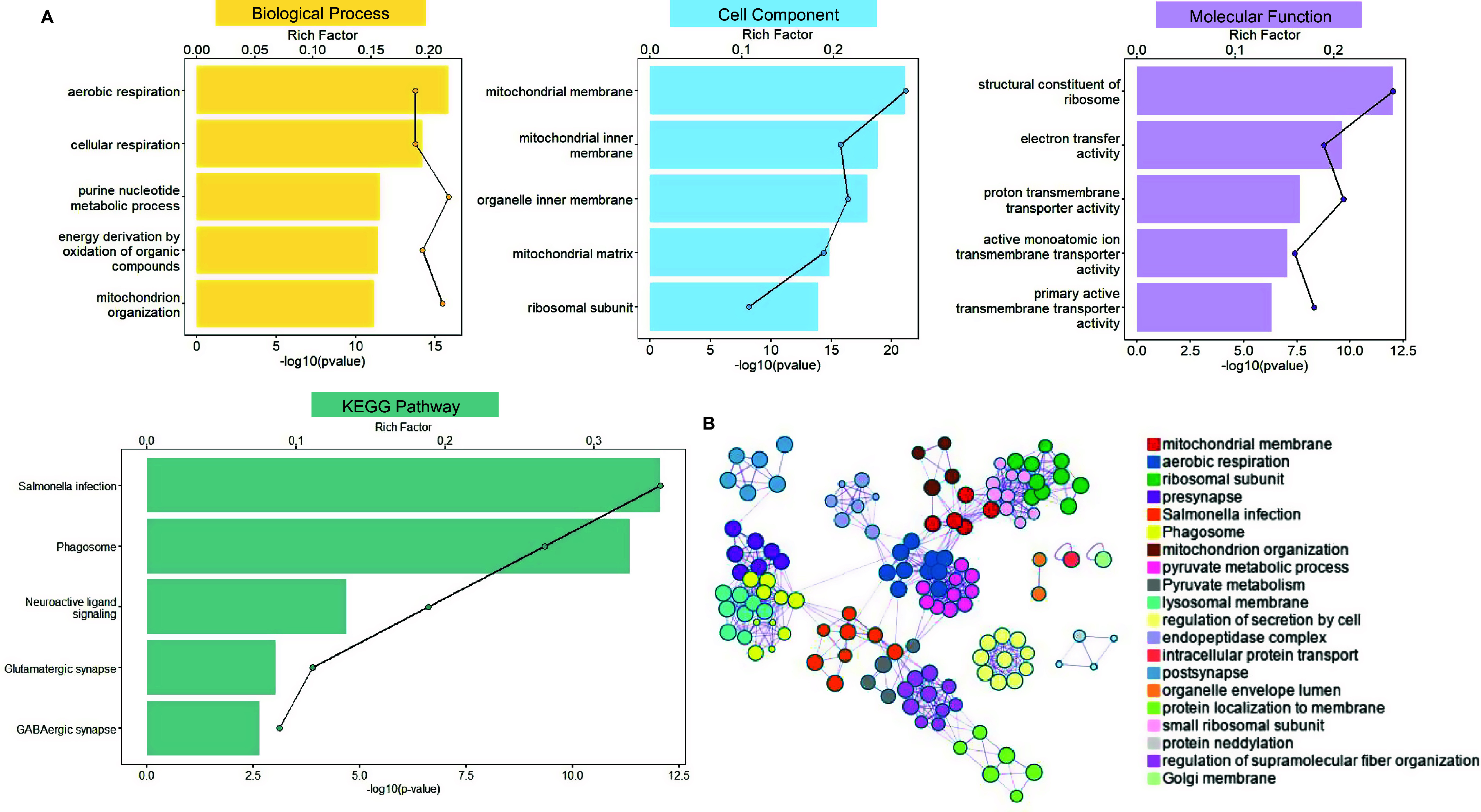
ADHD-C (A/B) functional enrichment of PLS1+ genes across GO and KEGG categories and pathways. (A) Bar colors represent the -log₁₀ (Bonferroni-corrected *p*-value) of enrichment for PLS1+ genes in Gene Ontology categories – biological processes (yellow), molecular functions (blue), cellular components (purple), and KEGG pathways (green). The black line indicates the count of PLS1+ genes significantly associated with each term. (B) Functional enrichment network of PLS1+ genes generated by Metascape. The network illustrates functional similarities among significantly enriched GO terms and KEGG pathways. Node size corresponds to the number of genes within each term; edges reflect functional relatedness between terms. Colors indicate distinct clusters of biologically coherent themes. *Note:* ADHD-C, attention-deficit/hyperactivity disorder combined type; PLS, partial least squares. GO, Gene Ontology; KEGG, Kyoto Encyclopedia of Genes and Genomes.

GO and KEGG enrichment analyses highlighted distinct functional profiles for PLS+/− genes in ADHD-C. PLS+ genes were primarily enriched in biological processes related to energy metabolism, such as mitochondrial organization and aerobic respiration; cellular components, including mitochondrial membranes and ribosomal subunits; and molecular functions, including phosphorylation and G-protein-coupled receptor signaling. KEGG pathway analysis further identified enrichment in glutamatergic synapse, neuroactive ligand-receptor interaction, and GABAergic synapse pathways (Figure 5A,B).

**Figure 6.**
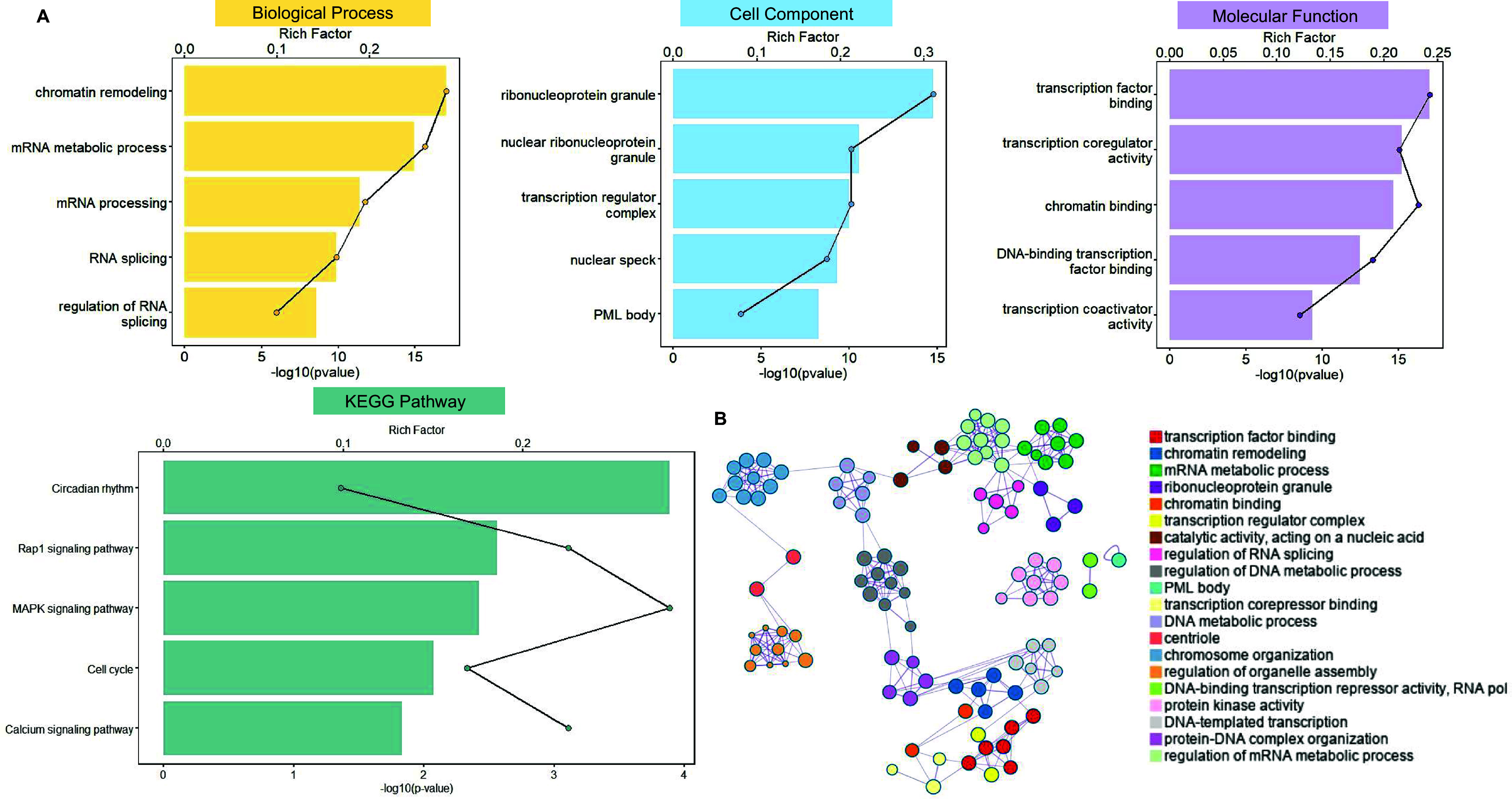
ADHD-C (A/B) functional enrichment of PLS1− genes across GO and KEGG categories and pathways. (A) Bar colors represent the −log₁₀ (Bonferroni-corrected *p*-value) of enrichment for PLS1+ genes in Gene Ontology categories – biological processes (yellow), molecular functions (blue), cellular components (purple), and KEGG pathways (green). The black line indicates the count of PLS1− genes significantly associated with each term. (B) Functional enrichment network of PLS1− genes generated by Metascape. The network illustrates functional similarities among significantly enriched GO terms and KEGG pathways. Node size corresponds to the number of genes within each term; edges reflect functional relatedness between terms. Colors indicate distinct clusters of biologically coherent themes. *Note:* ADHD, attention-deficit/hyperactivity disorder; PLS, partial least squares; GO, Gene Ontology; KEGG, Kyoto Encyclopedia of Genes and Genomes.

In contrast, PLS– genes were primarily enriched in biological processes involved in the regulation of gene expression, including messenger ribonucleic acid (mRNA) metabolic processes and RNA splicing. Their associated cellular components included nucleic acid–processing structures, such as ribonucleoprotein granules and chromatin, while molecular functions encompassed transcription factor binding and deoxyribonucleic acid (DNA)-binding transcription repressor activity. KEGG pathway analysis highlighted enrichment in circadian rhythm, Ras association proximate 1 (Rap1) signaling, mitogen-activated protein kinase (MAPK) signaling, and calcium signaling pathways (Figure 6A,B). These findings indicate a clear functional distinction between PLS+ and PLS– genes in ADHD-C: PLS+ genes are mainly involved in cellular energy metabolism and organelle homeostasis, whereas PLS– genes predominantly contribute to gene expression regulation and neural signaling pathways.

### ADHD risk genes and PLS1 networks share functions in neuronal and synaptic Pathways

To further examine the relationship between PLS1+/− gene sets and ADHD polygenic risk identified in GWAS (Demontis et al., 2023; Franke, Neale, & Faraone, 2009; Li et al., 2017), we conducted a multi-gene-set enrichment analysis using the Metascape platform. This analysis revealed significant overlap between ADHD GWAS risk genes and PLS1+/− genes from both the full ADHD cohort and the ADHD-C subtype (Supplementary Figures R5A, R6A). Functional annotation using Metascape further showed that the pathways enriched in PLS1+/− genes were largely overlapping between the full ADHD cohort and the ADHD-C subtype (Supplementary Figures R5B, R6B). In contrast, only a subset of PLS1+ gene terms showed partial overlap with pathways associated with ADHD risk genes, including postsynapse and pyruvate metabolic processes. On the other hand, PLS1− genes were enriched only in the chromatin remodeling pathway (Supplementary Figures R5C, R6C).

### PLS1+/− genes stratify distinct cortical layers and are enriched in excitatory and inhibitory neurons

To further refine our analysis and address cellular heterogeneity in the brains of individuals with ADHD and ADHD-C, PLS1+ and PLS1− genes were mapped to seven major cell types using an indirect annotation approach: Neuno-Ex, Neuno-In, astrocytes, microglia, oligodendrocytes, OPCs, and endothelial cells.

**Figure 7.**
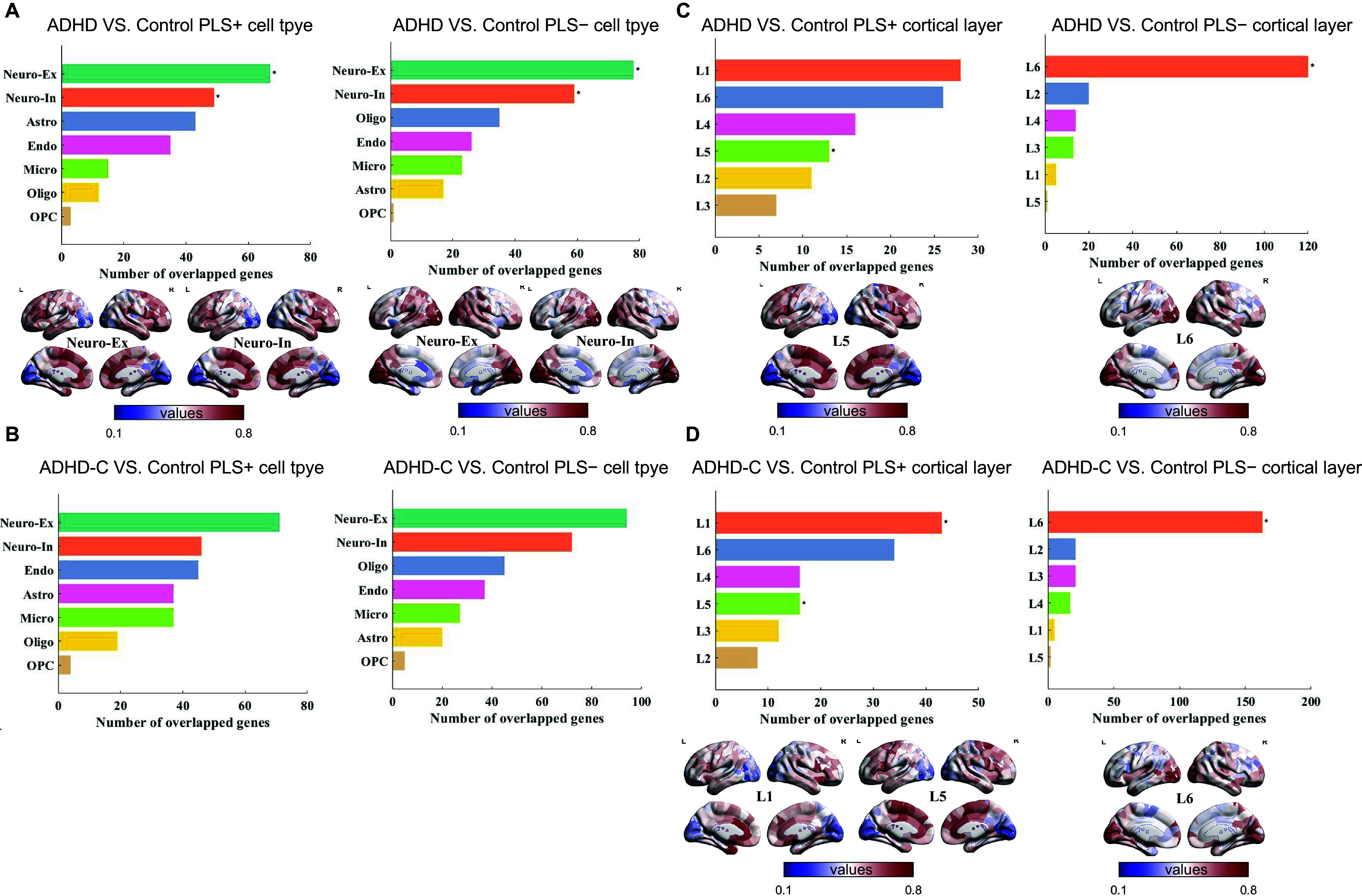
Cell type and cortical layer enrichment of PLS1+/− genes in ADHD and ADHD-C compared to TD. (A) ADHD versus TD: (left) Number of overlapping PLS1+ genes across cell types, with significant enrichment in inhibitory (number = 49, *p*_FDR_*_*_perm_ = 0.048) and excitatory neurons (number = 67, *p*_FDR_*_*_perm_ = 0.025); (right) PLS1− genes show significant enrichment in inhibitory (number = 59, *p*_FDR_*_*_perm_ = 0.024) and excitatory neurons (number = 78, *p*_FDR_*_*_perm_ = 0.024). Regional expression maps are shown for each gene set. Regional expression maps are shown for the overlapping gene sets. (B) ADHD-C versus TD: (left) No significant enrichment of PLS1+ genes in any cell type after multiple-testing correction (all *p*_FDR_*_*_perm_ > 0.05). (right) PLS1− genes show trend-level enrichment in excitatory (n = 94, *p*_FDR_*_*_perm_ = 0.069) and inhibitory (*n* = 72, *p*_FDR_*_*_perm_ = 0.069) neurons. (C) ADHD versus TD – cortical layers: GSEA revealed that PLS1+ genes were significantly enriched in cortical layer V (left, *p*_FDR_*_*_perm_ = 0.001); while PLS1− genes showed significant enrichment in layer VI (right, *p*_FDR_*_*_perm_ = 0.001). Regional expression maps are shown for the overlapping gene sets. (D) ADHD-C versus TD – cortical layers: GSEA revealed that PLS1+ genes were significantly enriched in cortical layers I and V (left, both *p*_FDR_*_*_perm_ = 0.001), while PLS1− genes showed significant enrichment in layer VI (right, *p*_FDR_*_*_perm_ = 0.001). Regional expression maps are shown for the overlapping gene sets. *Note:* ADHD, attention-deficit/hyperactivity disorder combined type; ADHD-C, attention-deficit/hyperactivity disorder combined type; TD, typically developing; *p*_FDR_, *p*-values after false discovery rate correction; *p*_perm_, permutation test; PLS, partial least squares.

We observed that PLS1+ and PLS1− genes were significantly associated with specific neuronal populations. In the full ADHD cohort, both gene sets were enriched in Neuno-In (PLS+: n = 49, *p*_FDR_*perm*_ = 0.048, PLS−: n = 59, *p*_FDR_*perm*_ = 0.024) and Neuno-Ex (PLS+: n = 67, *P*_FDR_*_perm* = 0.025; PLS−: n = 78, *p*_FDR_*perm*_ = 0.024) (Figure 7A). In ADHD-C, Neuno-Ex and Neuno-In also showed a trend toward enrichment for PLS1− genes, but these associations did not remain significant after full multiple-testing correction (Neuno-In: n = 72, *p*_FDR_*perm*_ = 0.074; Neuno-Ex: n = 94, *p*_FDR_*perm*_ = 0.076) (Figure 7B). Furthermore, cortical layer enrichment analysis showed that PLS1+ genes in the full ADHD cohort were predominantly enriched in layer V (*p*_FDR_*perm*_ = 0.001), whereas in ADHD-C, they were enriched in both layers I and V (*p*_FDR_*perm*_ = 0.001 for both). In contrast, PLS1− genes in both groups were primarily concentrated in layer VI (*p*_FDR_*perm*_ = 0.001) (Figure 7C,D).

### Gene expression patterns reveal divergent developmental trajectories

**Figure 8.**
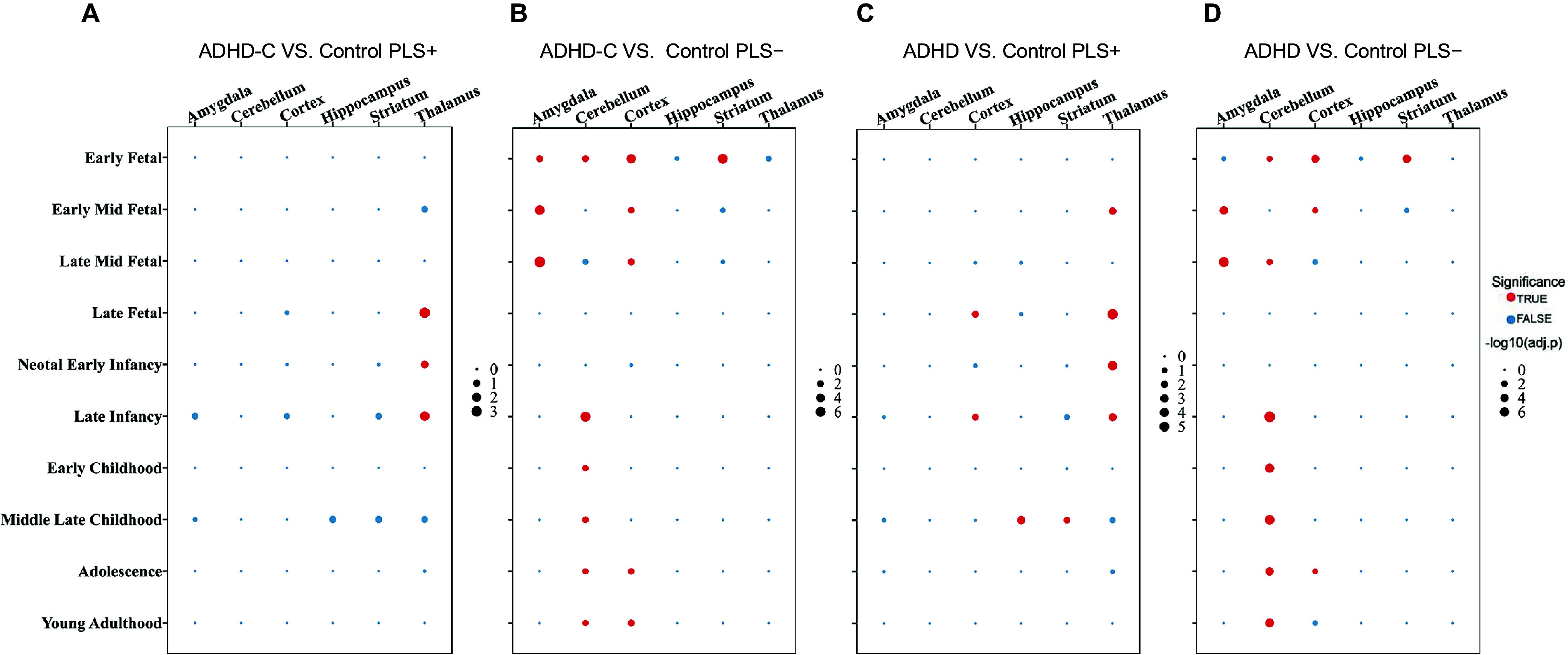
Developmental and regional enrichment patterns of PLS+ and PLS− genes. Dot plots show enrichment results across developmental stages and brain regions. Red dots indicate significant enrichment (*p*_FDR_ < 0.05), with dot size proportional to the enrichment strength (−log₁₀[p-value]); the value scale ranges from 0 to 6.00. Blue dots represent nonsignificant enrichment. (A) ADHD-C versus TD PLS1+; (B) ADHD-C versus TD PLS1−; (C) ADHD versus TD PLS1+; (D) ADHD versus TD PLS1 −. *Note:* ADHD, attention-deficit/hyperactivity disorder combined type; ADHD-C, attention-deficit/hyperactivity disorder combined type; TD, typically developing; *p*_FDR_, *p*-values after false discovery rate correction; PLS, partial least squares.

Analysis of developmental trajectories identified distinct spatiotemporal windows of gene expression specific to ADHD-C. PLS1+ genes exhibited persistent enrichment in the thalamus from the late fetal (LF) stage through late infancy (LI), indicating their potential role in early thalamic network formation and subsequent development of the limbic system, particularly in processes related to emotional memory integration (Figure 8A). In contrast, PLS1− genes showed significant expression in the amygdala and cortex from early to mid–late fetal stages, with pronounced cortical enrichment during adolescence and young adulthood. This pattern suggests their potential role in cortical remodeling and the regulation of emotional processes during puberty. In addition, PLS1− genes showed transient enrichment in the cerebellum and striatum during early fetal stages, followed by sustained high expression in the cortex from LI through young adulthood. This pattern indicates a potential role in the maturation of cerebellar-mediated motor and cognitive networks, which could have a significant impact on ADHD-C pathogenesis (Figure 8B).

Notably, both the full ADHD cohort and the ADHD-C subtype showed highly consistent developmental trajectories relative to TD controls, with significant alterations observed in PLS1+/− genes in both groups (Figure 8C,D). The overlapping trajectories indicate that the spatiotemporal gene expression patterns in ADHD-C capture broader neurodevelopmental abnormalities associated with ADHD. These findings highlight that ADHD-C embodies core neurobiological features of the disorder and provide valuable insights into its shared pathological mechanisms.

### MIND alterations are robust across multiple validation analyses

Overall, our primary findings were highly robust across multiple sensitivity and validation analyses. Key consistencies included: (i) a strong spatial correspondence between case–control MIND differences with and without TIV adjustment (ADHD: *r* = 0.88, *p*_spin_ < 0.001; ADHD-C: *r* = 0.996, *p*_spin_ < 0.001; Supplementary Figure R7); (ii) after controlling for symptom severity and FSIQ, only ADHD-C exhibited significant regional MIND differences compared with TD, with affected brain regions largely overlapping those identified in the main analysis (Supplementary Table R5); (iii) stability of case–control MIND difference maps across a range of connection densities, in both the full ADHD group *(r* = 0.13 ~ 0.84, *p* < 0.05 except at 50%: *p*_spin_ = 0.272; Supplementary Figure R8) and the ADHD-C subgroup *(r = −*0.15 ~ 0.69, all *p*_spin_ < 0.05 except at 30%: *p*_spin_ = 0.067; Supplementary Figure R9); (iv) high reproducibility of the ADHD-related MIND alterations in a large, independent external validation cohort aggregated from four datasets (ADHD, 12.93 ± 3.27 years old, 102 males; TD, 13.40 ± 3.98 years old, 25 males). This was evidenced by significantly divergent frequency distributions of regional MIND differences between ADHD and TD (*p* = 0.006) and an exceptionally strong spatial correlation between the case–control *t*-maps of the discovery and validation cohorts (*r* = 0.334, *p*_spin_ < 0.001, Supplementary Figure R10); (v) strong concordance of MIND values between the Schaefer 400 and DK308 atlases in the ADHD (*r* = 0.997, *p* < 0.001) and control groups (*r* = 0.995, *p* < 0.001), with highly consistent regional and network-level differences across both atlases (Supplementary Figures R11 and R12; Supplementary Tables R6–R8); and (vi) significant overlap in GO terms and pathways between ADHD-related GWAS genes and PLS1+/− genes. Collectively, these findings confirm that the identified epicenter patterns are robust and not dependent on the choice of cortical parcellation scheme.

## Discussion

This study investigated abnormal cortical structural similarity in ADHD using the MIND network, with a focus on the ADHD-C subtype. Case–control MIND alterations were spatially correlated with cortical gene expression patterns captured by the PLS1 score. PLS1+ genes – those most strongly associated with MIND changes – were predominantly enriched in metabolism-related pathways and exhibited clear cortical layer (layer V) and developmental stage (LF to LI) specificity. While PLS1+ genes were broadly expressed across regions and stages, significant enrichment in neuronal cell types (Neuro-Ex, Neuro-In) was observed only in the full ADHD cohort. Notably, substantial overlap in PLS1-related genes and functional pathways was found between the full cohort and ADHD-C, suggesting shared core pathological mechanisms. These findings link macroscale structural abnormalities with transcriptional regulation in ADHD, offering new insights into disease pathogenesis and progression.

The MIND index captures regional variations in multidimensional morphological features and reflects cortical structural similarity, offering a robust measure of individual differences in brain connectomes shaped by genetic and developmental factors (Sebenius et al., 2023. In this study, we found a significant global increase in MIND values in ADHD patients, particularly in temporal and parietal regions. This elevation indicates greater morphological homogeneity across cortical regions, reflecting reduced regional specialization – a phenomenon consistent with cortical dedifferentiation and insufficient segregation of large-scale functional networks. Such patterns have also been reported in other neurodevelopmental and psychiatric disorders, including schizophrenia and MDD (Xue et al., 2025; Yao et al., 2024), where they are interpreted as markers of developmental immaturity or delayed synaptic pruning. These abnormalities extended across multiple functional networks, supporting the view that ADHD pathophysiology involves widespread multisystem dysregulation rather than isolated regional deficits (Bode et al., 2015; Cao, Shu, Cao, Wang, & He, 2014; Michelini et al., 2019; Thapar & Cooper, 2016).

Subtype analyses further revealed that the spatial distribution of case–control MIND differences in ADHD-C closely mirrored that of the full ADHD cohort, but with greater spatial coverage and stronger effect sizes, a pattern not observed in the ADHD-I group. These results indicate that the ADHD-C subtype is associated with more pronounced cortical structural alterations and greater neurodevelopmental deviation. Supporting this, previous studies have reported reduced hippocampal volume in ADHD-C compared with ADHD-I (Al-Amin, Zinchenko, & Geyer, 2018), and other research has demonstrated that the ADHD-I group exhibits intermediate morphological features in the caudate nucleus relative to TD controls and ADHD-C (Vilgis, Sun, Chen, Silk, & Vance, 2016). These findings indicate that ADHD-I may represent a relatively milder phenotype. Considering its less pronounced neuroanatomical alterations and clinical symptoms, ADHD-I could differ qualitatively from ADHD-C in terms of neurodevelopmental deviation. Consequently, the subsequent analyses in this study will focus on the full ADHD cohort and the ADHD-C subtype, as these groups consistently capture the characteristic neuroanatomical changes and genetic foundations of the disorder.

MIND abnormalities showed no significant association with clinical symptoms in either the full ADHD cohort or the ADHD-C subtype. This dissociation suggests that MIND alterations may represent a stable neurobiological vulnerability (trait) rather than a state-dependent marker. Consequently, the clinical manifestation of symptoms likely emerges from the interaction between this structural backbone and other unmeasured environmental or cognitive factors, serving as an endophenotypic marker independent of current symptom severity. However, given that the current dataset lacked detailed measures of environmental influences and cognitive resilience, we were unable to directly quantify how these variables modulate the impact of MIND-based vulnerability on symptom severity. Future longitudinal studies incorporating comprehensive environmental and cognitive assessments are therefore warranted to disentangle these complex interactions. Moreover, our reproducibility analysis demonstrated that the case–control differential *t* values remained stable across variations in TIV, connection density, and data source, thereby confirming the robustness of our findings.

The widespread elevation of regional MIND values in ADHD-C reflects a state of excessive morphological homogeneity, which we postulate imposes a heightened metabolic demand on neural tissues to maintain such an extensive network state. Aligning with this bioenergetic hypothesis, our transcriptomic analysis revealed that PLS1+ genes were significantly enriched in mitochondrial respiration and cellular metabolism pathways. This resonates with established evidence linking mitochondrial dysfunction to neurodevelopmental disorders, where compromised bioenergetics impair critical processes such as neurotransmitter release, synaptic plasticity, and neuronal growth (Al-Amin et al., 2018; Satterstrom et al., 2020; Yokokura et al., 2021). Specifically, NT5DC3 serves as a critical link; it plays a role in nucleotide metabolism and is located within a genome-wide significant ADHD risk locus (rs11420276) identified by GWAS (Demontis et al., 2019). This suggests that metabolic dysregulation, potentially driven by NT5DC3, may underlie the bioenergetic burden of inefficient cortical networks.

It is important to acknowledge that metabolic dysfunction is likely a trans-diagnostic feature shared across psychiatric disorders, including ASD and schizophrenia (Purcell et al., 2023). However, we propose that in the specific biological context of ADHD, this metabolic vulnerability interacts with disorder-specific structural constraints to impair the development of these functionally costly, morphologically homogenized networks, particularly affecting the high-energy Layer V pyramidal neurons.

Conversely, PLS1− genes were enriched in neuroactive ligand-receptor interaction and circadian rhythm pathways, highlighting disruptions in synaptic plasticity and dopaminergic signaling. Dysregulation in the binding of neuroactive ligands, such as dopamine and norepinephrine, to their receptors can impair prefrontal control over subcortical regions like the basal ganglia and locus coeruleus, contributing to core symptoms such as inattention, hyperactivity, and impulsivity (Volkow et al., 2009). Notably, SEMA6D, which regulates axon guidance and synaptic connectivity, showed the strongest negative correlation. SEMA6D is located within one of the earliest identified ADHD risk loci (Demontis et al., 2019) and contributes to embryonic axon guidance, reinforcing the link between structural connectivity deficits and synaptic signaling. Furthermore, our findings align with the role of circadian disruption in ADHD. Beyond established associations with CLOCK gene variants (Kissling et al., 2008; Xu et al., 2010), a recent Mendelian randomization study demonstrated that delayed sleep timing is causally associated with an approximate 8-fold increase in ADHD risk (Dai et al., 2025). This indicates that circadian dysregulation may act as a mechanism exacerbating synaptic deficits.

Cellular abnormalities and the organization of cortical neurons play a critical role in the development of various psychiatric disorders, including bipolar disorder (BD), schizophrenia, autism spectrum disorder (ASD), and MDD (Alexander-Bloch et al., 2018). Through matched analyses incorporating cell-type, our results provide structural evidence for the ‘Excitation/Inhibition (E/I) Imbalance’. model. In the full ADHD cohort, PLS1+/− genes associated with MIND exhibit substantial enrichment in Neuno-In and Neuno-Ex neuronal populations. However, the PLS1− genes linked to ADHD-C exhibited a pronounced tendency to overlap with genes expressed in both neuronal categories. Recent studies have shown dysregulation in both excitatory and inhibitory pathways in individuals with ADHD. This excitation–inhibition imbalance model has been corroborated by research in both human brains and animal models (Morgan et al., 2010; Satterstrom et al., 2020; Suzuki et al., 2013; Yokokura et al., 2021). Crucially, PLS1+ genes were predominantly enriched in cortical Layer V, which contains pyramidal tract neurons (PTNs) – the principal motor output pathway. Recent super-resolution mapping reveals that Layer V PTNs undergo critical dendritic spine remodeling during adolescence to form functional ‘hotspots’ required for signal integration (Egashira et al., 2026). The enrichment of metabolic and synaptic genes (PLS1+) in this layer suggests that this high-cost developmental remodeling is vulnerable in ADHD. We propose that molecular dysregulation in these primary motor output neurons impairs the filtration of motor commands, directly underlying the motor disinhibition and hyperactivity characteristic of the ADHD-C subtype. Furthermore, the involvement of deep layers (V and VI) aligns with recent cross-species findings that these layers drive cortical morphological changes and inhibitory regulation (Liu et al., 2025), confirming deep-laminar vulnerability as a core feature of ADHD pathology.

Furthermore, our developmental trajectory analysis suggests that these abnormalities are rooted in specific spatiotemporal windows. PLS1+ genes were continuously enriched in the thalamus from the LF to LI stages, indicating their potential role in the development of early thalamocortical circuits. These circuits deliver critical input to the maturing cortex during the latter half of gestation and are essential for establishing lifelong sensory integration networks (Li et al., 2013; O’Leary, Chou, & Sahara, 2007; Sharma, Angelucci, & Sur, 2000; Sur & Rubenstein, 2005; Sydnor et al., 2025; Vue et al., 2013). Previous studies have reported delayed cortical maturation in children with ADHD (Shaw et al., 2007). Importantly, individuals with the ADHD-C subtype exhibit reduced subcortical volume (Mu, Wu, Zhang, & Chang, 2022), and meta-analytic evidence indicates that structural differences in subcortical regions are most pronounced in children with ADHD, with less marked effects in adults (Hoogman et al., 2017). Our findings further imply that early disruptions in thalamocortical development may constitute a core pathogenic mechanism underlying ADHD.

In contrast, PLS1− genes exhibited a biphasic expression profile. During the fetal period, they were co-enriched in the amygdala, cortex, striatum, and cerebellum, aligning with previous findings that gene expression during key developmental windows plays a crucial role in shaping amygdala volume (Ji et al., 2025). Notably, PLS1− genes were re-expressed in the cortex and cerebellum during adolescence, corresponding with periods of peak synaptic pruning (Hua & Smith, 2004; Katz & Shatz, 1996) and reorganization of the monoaminergic system (O’ O’Leary et al., 2014, Pitzer, 2019). The cerebellum is essential for motor coordination and contributes to cognitive and emotional processes. Longitudinal research on children with ADHD aged 7–19 years has linked reduced cerebellar volume to deficits in attentional control and behavioral inhibition (Chang, Lin, & Gau, 2024). These findings indicate that ADHD pathophysiology involves more than the traditional prefrontal–striatal circuitry, highlighting the cerebellum as a promising target for therapeutic intervention.

Finally, while our study focused on structural networks in ADHD, emerging evidence from static and dynamic functional connectivity studies offers complementary mechanistic insights into psychiatric disorders (Li et al., 2025; You et al., 2022). Specifically, (Li et al., 2025) demonstrated aberrant frontolimbic functional connectivity in ADHD youth with familial risk, while (You et al., 2022) identified disrupted dynamic functional states in schizophrenia. Given that our MIND-based analysis revealed morphologically homogenized structural networks, we speculate that such structural alterations may constrain the temporal flexibility of dynamic functional states. Future multimodal studies integrating structural and functional connectivity are warranted to elucidate the structure–function coupling underlying ADHD symptomatology.

### Limitation

Several limitations should be noted. First, the MIND networks were derived from five morphological features; future work should incorporate broader microstructural measures to capture more comprehensive brain alterations. Second, our transcriptomic analyses relied on the AHBA dataset, which comprises adult, postmortem, predominantly left-hemisphere samples. This introduces important considerations for interpreting our PLS results. The developmental mismatch between the transcriptional architecture captured in AHBA and the age-specific regulatory networks active in our pediatric ADHD cohort may limit the precision of PLS weighting and trajectory interpretations. While previous work(Li, Chen, & Lin, 2021; Sebenius et al., 2023) has supported the relevance of the identified genes to neurodevelopmental windows, dynamic shifts in gene co-expression during development cannot be ruled out. Moreover, recent evidence of gene expression differences between living and postmortem brains (Liharska et al., 2026) suggests that postmortem intervals may alter specific metabolic signatures. Regarding hemispheric asymmetry, although prior studies (Li, Chen, & Lin, 2021, Li, Li, et al., 2025, Niu et al., 2026, Sebenius et al., 2023 (Arnatkeviciute, Fulcher, & Fornito, 2019) indicate that cortical gene expression patterns are sufficiently conserved across hemispheres to support cross-hemispheric inference, subtle asymmetries may still influence results. Future studies incorporating age-matched, living-brain transcriptomic data are therefore warranted.

Third, the absence of a psychiatric control group (e.g. ASD) limits our ability to assess diagnostic specificity. Although we hypothesize that metabolic dysfunction interacts with ADHD-related structural phenotypes, mitochondrial deficits are inherently transdiagnostic; comparative studies will be essential to distinguish disorder-specific molecular signatures from shared vulnerabilities. Fourth, due to the small sample size of the ADHD-H subtype (n = 6), these participants were excluded from primary analyses. Consequently, our findings may not generalize to individuals with the pure hyperactive/impulsive presentation. Future studies with larger ADHD-H samples are needed to determine whether similar MIND alterations occur in this subgroup. Finally, the cross-sectional design and lack of detailed environmental data preclude causal inferences about the interplay between neurobiological vulnerability and symptom severity.

## Conclusion

This study shows that the MIND framework reliably identifies abnormal patterns of cortical similarity in ADHD, particularly within ADHD-C. Regional MIND alterations exhibited a strong spatial alignment with cortical gene expression profiles, predominantly influenced by PLS1. PLS1+ genes were primarily enriched in metabolic pathways, displayed cortical layer specificity (notably in layer V), and were developmentally regulated from late fetal to early childhood stages. Both the full ADHD cohort and the ADHD-C subtype shared a core set of PLS1-related genes and overlapping functional pathways, indicating shared underlying neurobiological mechanisms. These multilevel results connect large-scale cortical structural alterations with fine-scale transcriptional regulation, emphasizing common pathological mechanisms across different ADHD phenotypes.

## Supporting information

10.1017/S003329172610405X.sm001 Zeng et al. supplementary material Zeng et al. supplementary material

## Data Availability

The raw neuroimaging and phenotypic data used in this study are publicly available via the **ADHD-200 Consortium** (http://fcon_1000.projects.nitrc.org/indi/adhd200/) and **OpenNeuro** (Accession Numbers: ds002424, ds005899, ds004605). The transcriptomic data resources are available at the following links: the **Allen Human Brain Atlas** (https://human.brain-map.org/static/download) and **BrainSpan** (https://www.brainspan.org/static/download.html). Regarding the analysis code, this study utilized established open-source pipelines. The code for calculating the **MIND networks** is available on GitHub (https://github.com/isebenius/MIND). The code for **Partial Least Squares (PLS) analysis** can be accessed at (https://github.com/SarahMorgan/Morphometric_Similarity_SZ). The scripts for **spatial permutation testing (spin tests)** are available at (https://github.com/frantisekvasa/rotate_parcellation). Any additional custom scripts or intermediate data used in this study are available from the corresponding author upon reasonable request.

## Supplementary material

The supplementary material for this article can be found at http://doi.org/10.1017/S003329172610405X.

## Data availability statement

The data included in this study are available from the corresponding author upon reasonable request.
