# Supplementary material for: Cortical morphometric inverse divergence in attention-deficit/hyperactivity disorder correlates with cell-type-specific, laminar-specific and developmental transcriptomic signatures
Source: Psychol Med. 2026 May 11;56:e136. doi: 10.1017/S003329172610405X (PMC13161819; doi:10.1017/S003329172610405X)
Supplement: Zeng et al. supplementary material [file S003329172610405Xsup001.docx]

**Supplementary Information**

[**Supplementary Methods (M) 2**](#_Toc84562759)

[S1. Participants 2](#_Toc1759599846)

[S2. Imaging acquisition parameters 6](#_Toc597308885)

[S3. MIND estimation and network construction 8](#_Toc1631864117)

[S4. Details of enrichment analyses 9](#_Toc1226558582)

[**Supplementary Result (R) Figures 11**](#_Toc1074560121)

[sFig. R1 Assessment of covariate balance before and after propensity score matching. 11](#_Toc1941966024)

[sFig. R2 Evaluation of ComBat harmonization effectiveness using Principal Component Analysis (PCA). 12](#_Toc1166498262)

[sFig. R3 Variance in case-control MIND differences explained by PLS components in full ADHD cohort 13](#_Toc958075971)

[sFig. R4 Association Between PLS1+/– Genes and Psychiatric Disorder Genes 14](#_Toc550459391)

[sFig. R5 PLS1+ Multi-gene Set Enrichment Analysis of ADHD-associated Transcriptomic Profiles 15](#_Toc211433261)

[sFig. R6 PLS1– Multi-gene Set Enrichment Analysis of ADHD-associated Transcriptomic Profiles 16](#_Toc1620865489)

[sFig. R7 Validation of the effect of TIV on case–control MIND differences 17](#_Toc1056211428)

[sFig. R8 Replicability of MIND across connection densities in case (ADHD) - control *t* values 18](#_Toc645644294)

[sFig. R9 Replicability of MIND across connection densities in case (ADHD-C) - control *t* values 19](#_Toc108780967)

[sFig. R10 Reproducibility of ADHD-related MIND alterations in an independent external validation cohort. 20](#_Toc773128772)

[sFig. R11 Schaefer 400 Atlas Reveals Altered MIND Patterns in ADHD and Its Subtypes 21](#_Toc1699206654)

[sFig. R12 Cross-Atlas Reliability of MIND Values 22](#_Toc1328695972)

### **Supplementary Methods (M)**

#### **S1. Participants**

In the present study, we utilized data from four sites: Peking University (PKU), New York University Child Study Center (NYU), Oregon Health & Science University (OHSU), and Kennedy Krieger Institute (KKI). The dataset included both individuals with attention-deficit/hyperactivity disorder (ADHD) and typically developing (TD) controls, comprising 257 patients and 263 controls, totaling 520 participants. Intelligence quotient (IQ) and ADHD symptom measures were collected across all sites, though assessment tools varied by site: the Wechsler Intelligence Scale for Chinese Children-Revised was used at PKU, the Wechsler Abbreviated Scale of Intelligence at NYU, and the Wechsler Intelligence Scale for Children at KKI. ADHD symptoms were assessed using the ADHD Rating Scale IV at PKU, the Conners’ Parent Rating Scale Revised (Long version) at NYU and KKI, and the Conners’ Rating Scale–3rd Edition at OHSU (**sTable M1**).

Following quality control of T1-weighted magnetic resonance imaging data and preprocessing steps, several participants were excluded due to data quality issues. Specifically, 4 participants from NYU were excluded based on T1 quality, with an additional 13 excluded during preprocessing for MIND analysis; 14 from PKU based on T1 quality; 3 from KKI based on T1 quality, plus 1 during preprocessing; and 4 from OHSU during preprocessing. After these exclusions, 481 participants remained, including 212 with ADHD and 269 TD controls.

To mitigate potential confounding effects, propensity score matching was applied using gender and age as covariates in a logistic regression model. Matching was performed using a caliper width of 0.073. This process yielded a final matched sample of 176 ADHD patients and 176 TD controls. Detailed demographic characteristics for the matched sample are provided in **sTable M2 and sTable M3**.

**sTable M1. Site-Specific Assessment Protocols and Inclusion Criteria**

| Category | NYU | Peking | KKI | OHSU |
| --- | --- | --- | --- | --- |
| Diagnostic Tools | KSADS-PL (parent/child interview) | C-DIS-IV + KSADS-PL (parent) | DICA-IV (parent interview) | KSADS-I (parent) + clinical review |
| Symptoms Scale | CPRS-R:LV (DSM-IV) | ADHD-RS IV | CPRS-R (DSM-IV) | Conners Scale |
| IQ Test | WASI (Full-scale IQ) | WISCC-R (Full-scale IQ) | WISC-IV (Index scores) | WISC-IV short form (3 subtests) |
| Washout Period | ≥24 hours | ≥48 hours (5 half-lives) | ≥24 hours | ≥5 half-lives (24-48 hours) |
| Inclusion Criteria | ADHD: KSADS-PL + CPRS-R T≥65 TD: No psychiatric disorder + CPRS-R T<60 | Right-handed No head trauma/neurological disease FSIQ>80 | ADHD: DICA-IV + CPRS-R T≥65/DuPaul criteria TD: No behavioral issues + CPRS-R T≤60 | ADHD: No neurological disorder/autism/ID TD: No conduct disorder/MDD/ADHD |

**sTable M2. Participant Inclusion and Exclusion by Site**

| Site | Initial participants | Excluded  (T1QC/Preprocessing) | Retained ADHD | Retained TD | Matched ADHD | Matched TD |
| --- | --- | --- | --- | --- | --- | --- |
| NYU | 222 | 14 | 112 | 93 | 86 | 62 |
| Peking | 136 | 17 | 44 | 78 | 39 | 41 |
| KKI | 83 | 4 | 21 | 58 | 18 | 43 |
| OHSU | 79 | 4 | 35 | 40 | 33 | 30 |
| Total | 520 | 39 | 212 | 269 | 176 | 176 |

notes: The retained ADHD cohort comprised 125 patients with the combined type (ADHD-C) and 87 with the inattentive type (ADHD-I). Following propensity score matching, the matched ADHD group included 105 ADHD-C and 71 ADHD-I patients.

**sTable M3. Demographic characteristics stratified by Site**

| Site | ADHD-C, n (%) | Age (years) | Male, n (%) | FSIQ |
| --- | --- | --- | --- | --- |
| **NYU** |  |  |  |  |
| ADHD (N = 86) | 56 (65.12) | 10.68 ± 2.70 | 61 (70.93) | 106.44 ± 12.73 |
| TD (N = 62) | / | 12.17 ± 3.12 | 43 (69.35) | 111.53 ± 14.17 |
| **Peking** |  |  |  |  |
| ADHD (N = 39) | 13 (33.33) | 10.94 ± 2.08 | 28 (71.79) | 103.85 ± 13.89 |
| TD (N = 41) | / | 10.55 ± 1.69 | 28 (68.29) | 119.71 ± 13.60 |
| **KKI** |  |  |  |  |
| ADHD (N = 18) | 4 (77.78) | 9.96 ± 1.57 | 8 (44.44) | 108.06 ± 15.19 |
| TD (N = 43) | / | 10.19 ± 1.27 | 32 (74.42) | 111.42 ± 10.62 |
| **OHSU** |  |  |  |  |
| ADHD (N = 33) | 22 (66.67) | 8.60 ± 0.81 | 23 (69.70) | 107.27 ± 13.74 |
| TD (N = 30) | / | 8.80 ± 1.08 | 17 (56.67) | 119.33 ± 12.49 |

notes: Values are presented as Mean ± SD.

#### **S2. Imaging acquisition parameters**

**Peking University (PKU)**

The dataset 1 images were acquired using a 3.0 T Siemens Discovery MR B15 MRI scanner. The acquisition parameters were configured as follows: 128 axial slices, field of view = 256 × 256 mm2, matrix = 256 × 192, voxel size = 1.3 ×1.0 × 1.3 mm, slice thickness = 1.33 mm (no gap), time echo =3.39 ms, time repetition = 2530 ms, flip angle = 7°.

The dataset 2 images were acquired using a 3.0 T Siemens Discovery MR B15 MRI scanner. The acquisition parameters were configured as follows: 176 axial slices, field of view = 256 × 208 mm2, matrix = 256 × 176, voxel size = 1.0 ×1.0 × 1.0 mm, slice thickness = 1.00 mm (no gap), time echo =3.45 ms, time repetition = 2530 ms, flip angle = 7°.

The dataset 3 images were scanned using 1 of 5 different sagittal T1-mprage protocols. The parameters are below. 192 slices, TR= 2000 ms, TE= 3.67 ms, Inversion Time= 1100 ms, slice thickness= 1 mm, Flip angle = 12°, FOV = 240×240, Matrix = 256×256 ; 128 slices, TR = 1950 ms, TE = 2.6 ms, Inversion Time = 900 ms, slice thickness= 1.3 mm, Flip angle = 10°, FOV = 240×256, Matrix = 240×256 ; 128 slices, TR = 2530 ms, TE = 3.37 ms, Inversion Time = 1100 ms, slice thickness = 1.33 mm, Flip angle = 7°, FOV = 256×256, Matrix = 256×256 ; 176 slices, TR = 1770 ms, TE=3.92 ms, Inversion Time = 1100 ms, slice thickness = 1 mm, Flip angle = 12°, FOV = 256×256, Matrix = 512×512 ; 144 slices, TR = 845 ms, TE = 2.89 ms, Inversion Time = 600 ms, slice thickness =1.3 mm, Flip angle＝ 8°, FOV = 261×261, Matrix = 256×256.

**New York University Child Study Center (NYU)**

The images were acquired using a 3.0 T Siemens MAGNETOM Allegra syngo MR 2004A MRI scanner. The acquisition parameters were configured as follows: 128 axial slices, field of view = 256 × 256 mm2, matrix = 256 × 192, voxel size = 1.3 ×1.0 × 1.3 mm, slice thickness = 1.33 mm (no gap), time echo =3.25 ms, time repetition = 2530 ms, flip angle = 7°.

**Kennedy Krieger Institute (KKI)**

The acquisition parameters were configured as follows: 200 axial slices, field of view = 256 × 200 mm2, matrix = 256 × 200, voxel size = 1.0 ×1.0 × 1.0 mm, slice thickness = 1.00 mm (no gap), time echo =3.7 ms, time repetition = 800 ms, flip angle = 8°.

**Oregon Health & Science University (OHSU)**

The images were acquired using a 3.0 T Siemens MAGNETOM TrioTim syngo MR B17 MRI scanner. The acquisition parameters were configured as follows: 160 axial slices, field of view = 256 × 240 mm2, matrix = 256 × 256, voxel size = 1.3 ×1.0 × 1.1 mm, slice thickness = 1.10 mm (no gap), time echo =3.58 ms, time repetition = 2300 ms, flip angle = 10°.

#### **S3. MIND estimation and network construction**

MIND was computed to estimate inter-regional morphometric similarity based on structural MRI data. For each individual, five vertex-wise morphometric features—gray matter volume (GMV), cortical thickness (CT), surface area (SA), mean curvature (MC), and sulcal depth (SD)—were standardized across the cortex and aggregated within each of 308 cortical regions defined by the Desikan–Killiany atlas. The similarity between the multivariate feature distributions of any two regions was quantified using the symmetric Kullback–Leibler divergence (Jeffrey’s divergence), which was then transformed into a bounded similarity metric ranging from 0 to 1, with higher values indicating greater similarity. This yielded a 308 × 308 MIND matrix per participant.

#### **S4. Details of enrichment analyses**

1. Cell Type Enrichment Analysis

We performed a cell type enrichment analysis to assess the specificity of PLS1-derived gene signatures across seven major brain cell types: astrocytes, endothelial cells, microglia, excitatory neurons, inhibitory neurons, oligodendrocytes, and oligodendrocyte precursor cells (OPCs). Marker genes were compiled from various human postmortem single-cell and single-nucleus RNA sequencing studies (Darmanis et al., 2015; Habib et al., 2017; Lake et al., 2018; Li et al., 2018; McKenzie et al., 2021; Zhang et al., 2016). We calculated the proportion of marker genes in the PLS1+/- sets and assessed statistical significance by comparing it to a null distribution generated from 10,000 random gene sets of the same size (Hansen et al., 2021).

2. Cortical Layer Enrichment Analysis

Additionally, we analyzed the laminar specificity of PLS1 gene expression using cortical layer marker genes from He et al. (He et al., 2017). The marker gene sets comprised 772 genes for layer I, 483 for layer II, 294 for layer III, 308 for layer IV, 115 for layer V, and 2,159 for layer VI. The enrichment analysis was conducted using the same statistical framework as the cell type analysis, comparing the observed proportion of layer marker genes in the PLS1+/- sets to the null distribution.

**References:**

Darmanis S, Sloan SA, Zhang Y, Enge M, Caneda C, and Shuer LM, et al. 2015.A survey of human brain transcriptome diversity at the single cell level. Proc. Natl. Acad. Sci. U. S. A. 112(23), 7285-7290.

Habib N, Avraham-Davidi I, Basu A, Burks T, Shekhar K, and Hofree M, et al. 2017.Massively parallel single-nucleus RNA-seq with DroNc-seq. Nat. Methods. 14(10), 955-958.

Hansen JY, Markello RD, Vogel JW, Seidlitz J, Bzdok D, and Misic B. 2021.Mapping gene transcription and neurocognition across human neocortex. Nat. Hum. Behav. 5(9), 1240-1250.

He Z, Han D, Efimova O, Guijarro P, Yu Q, and Oleksiak A, et al. 2017.Comprehensive transcriptome analysis of neocortical layers in humans, chimpanzees and macaques. Nat. Neurosci. 20(6), 886-895.

Lake BB, Chen S, Sos BC, Fan J, Kaeser GE, and Yung YC, et al. 2018.Integrative single-cell analysis of transcriptional and epigenetic states in the human adult brain. Nat. Biotechnol. 36(1), 70-80.

Li M, Santpere G, Imamura KY, Evgrafov OV, Gulden FO, and Pochareddy S, et al. 2018.Integrative functional genomic analysis of human brain development and neuropsychiatric risks. Science. 362(6420),

McKenzie AT, Wang M, Hauberg ME, Fullard JF, Kozlenkov A, and Keenan A, et al. 2021.Author Correction: Brain Cell Type Specific Gene Expression and Co-expression Network Architectures. Sci. Rep. 11(1), 19430.

Zhang Y, Sloan SA, Clarke LE, Caneda C, Plaza CA, and Blumenthal PD, et al. 2016.Purification and Characterization of Progenitor and Mature Human Astrocytes Reveals Transcriptional and Functional Differences with Mouse. Neuron. 89(1), 37-53.

### **Supplementary Result (R) Figures**


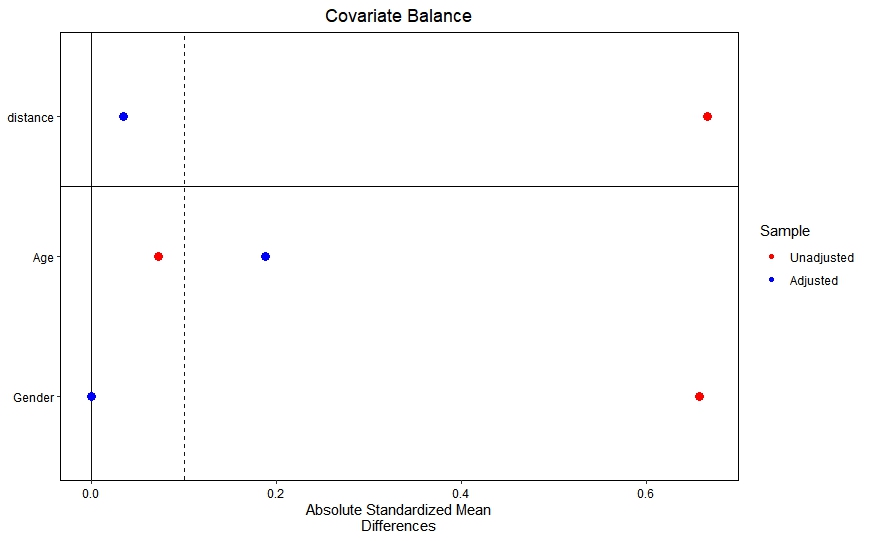


#### **sFig. R1 Assessment of covariate balance before and after propensity score matching.**

The Love plot displays the absolute standardized mean differences (SMD) for covariates (age and gender) between the ADHD and TD groups. The red dots represent the SMDs in the unmatched sample, while the blue dots represent the SMDs in the matched sample. The vertical dashed line indicates the threshold for acceptable balance (SMD < 0.1). Specifically, the SMD for sex was negligible (< 0.001), and the SMD for age was 0.191, falling within the acceptable range (< 0.2) for covariate balance.


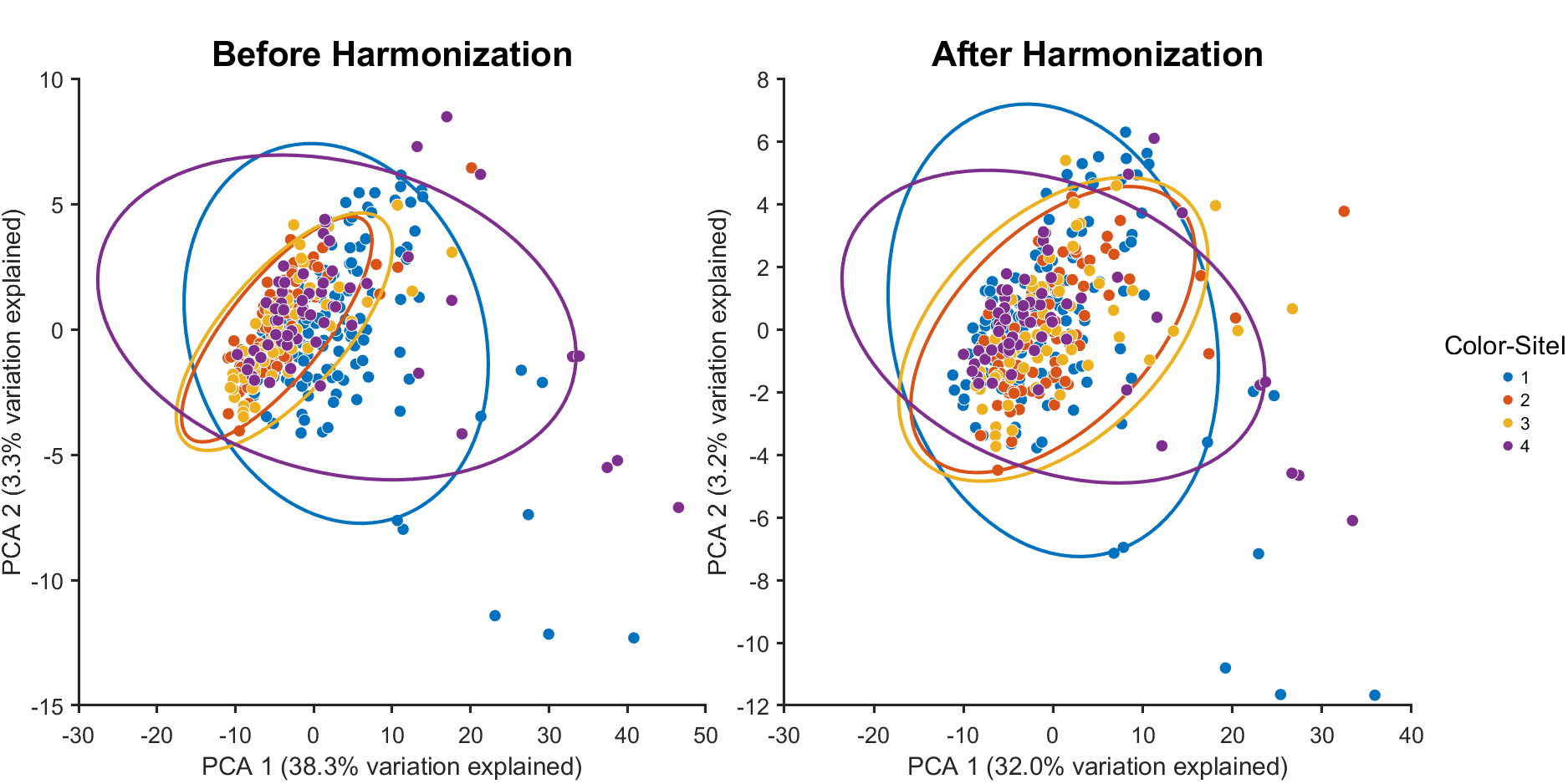


#### **sFig. R2 Evaluation of ComBat harmonization effectiveness using Principal Component Analysis (PCA).**

PCA plots show the distribution of individual subjects based on their regional MIND values across the whole brain, colored by scanning site. (A) Before ComBat correction: Distinct clusters corresponding to different scanning sites are visible, indicating substantial site-related variability. (B) After ComBat correction: Subjects from different sites are well-mixed, indicating that the site effects have been effectively removed while preserving the underlying biological structure of the data. Site 1: NYU; Site 2: PKU; Site 3: KKI; Site 4: OHSU.


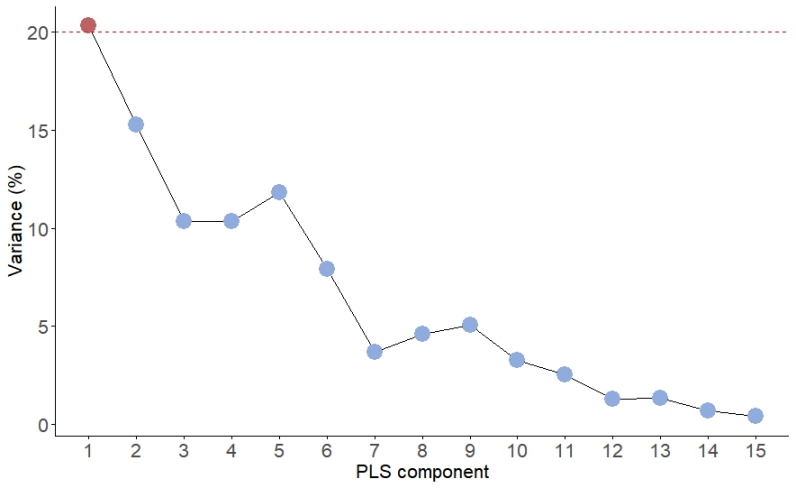


**

#### **sFig. R3 Variance in case-control MIND differences explained by PLS components in full ADHD cohort**

Variance in case–control MIND differences explained by the top 15 PLS components. Only the first component (PLS1) accounted for more than 20% of the variance (20.32%) and was statistically significant after controlling for spatial autocorrelation (*p*_spin_ = 0.005). ** *p* < 0.01.


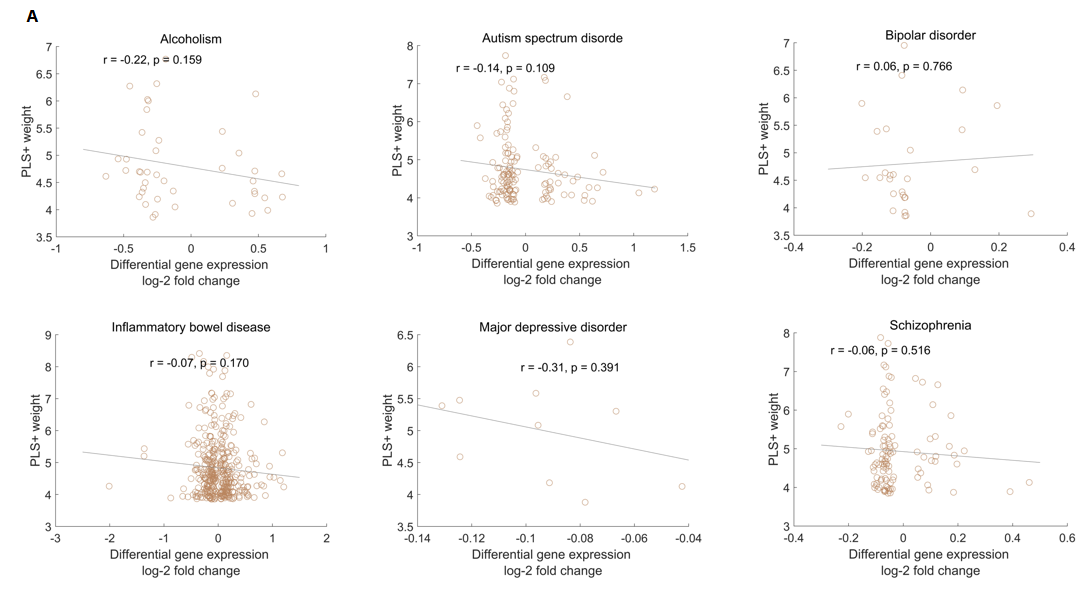


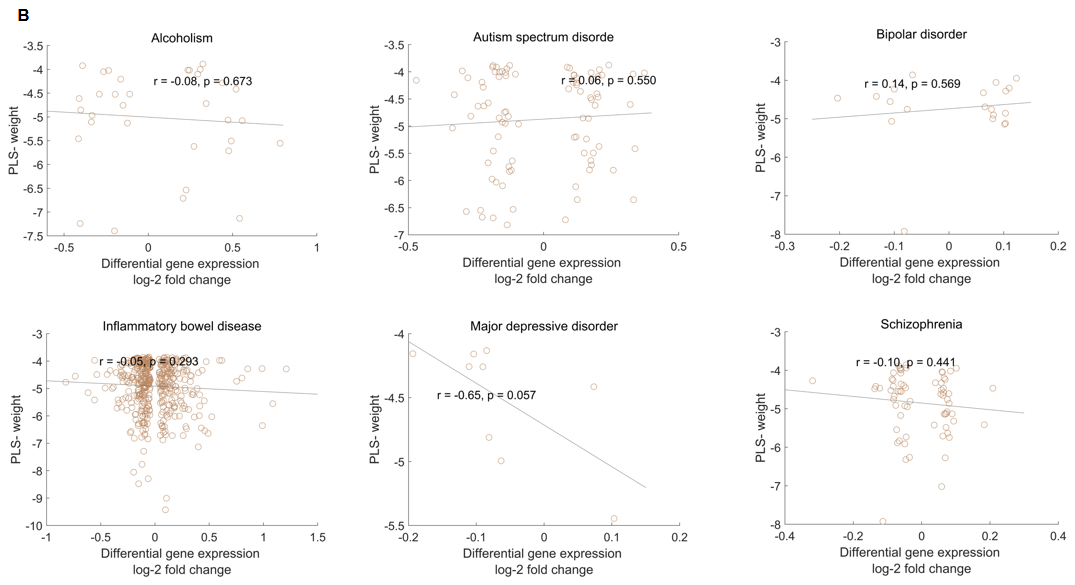


#### **sFig. R4 Association Between PLS1+/– Genes and Psychiatric Disorder Genes**

No significant association of PLS1+ (A) or PLS1– (B) genes with psychiatric disorder genes in the full ADHD cohort or ADHD-C subgroup (*P*_FDR_perm_ > 0.05).


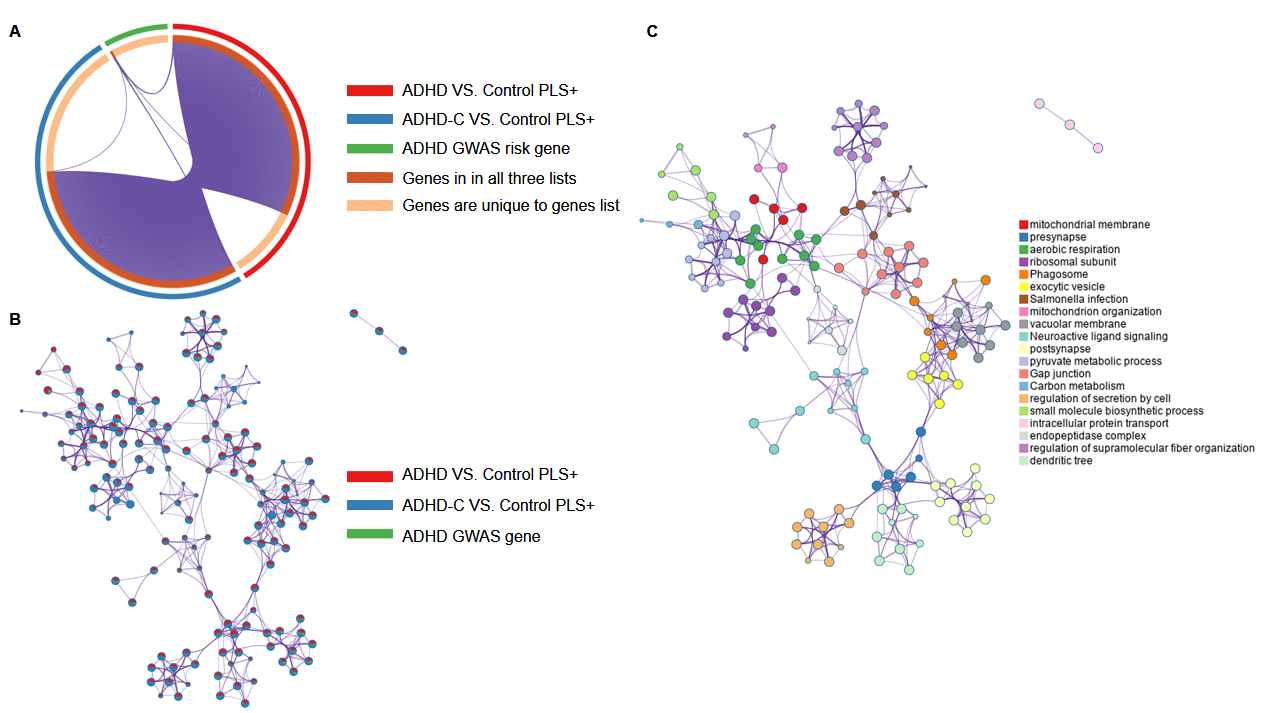


#### **sFig. R5 PLS1+ Multi-gene Set Enrichment Analysis of ADHD-associated Transcriptomic Profiles**

(A) Circos plot illustrating overlap among three gene sets: ADHD risk genes identified in prior GWAS (green), PLS1+ genes derived from the full ADHD cohort (red), and PLS1+ genes from the ADHD-C subtype (blue). Dark orange nodes represent genes shared across all sets; light orange nodes indicate genes unique to each set. Functionally related genes grouped under the same Gene Ontology (GO) term are connected by purple edges. (B) Functional enrichment analysis performed in Metascape. Significantly enriched pathways are displayed, with red dots denoting pathways enriched in ADHD PLS1+ genes, blue dots for ADHD-C PLS1+ genes, and green dots for GWAS-derived ADHD risk genes. All terms are *P*_FDR_ < 0.05. (C) Gene Ontology terms are labeled according to the color scheme of their associated gene set.


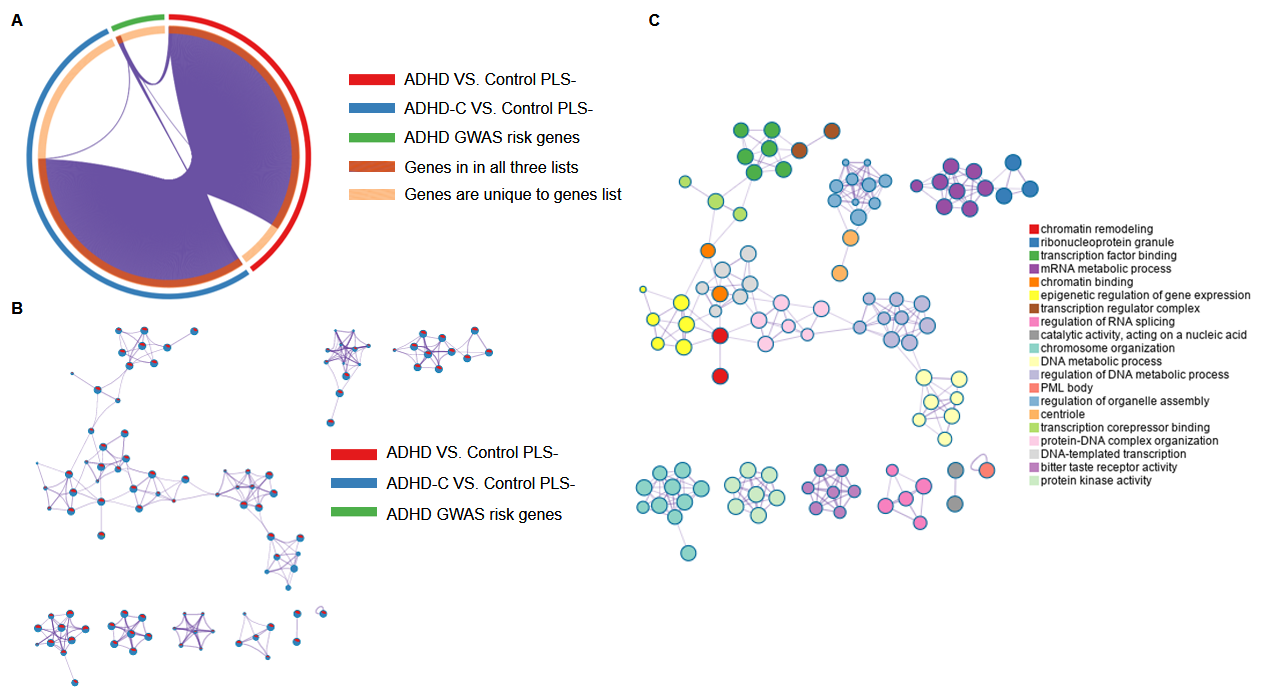


#### **sFig. R6 PLS1– Multi-gene Set Enrichment Analysis of ADHD-associated Transcriptomic Profiles**

(A) Circos plot illustrating overlap among three gene sets: ADHD risk genes identified in prior GWAS (green), PLS1- genes derived from the full ADHD cohort (red), and PLS1– genes from the ADHD-C subtype (blue). Dark orange nodes represent genes shared across all sets; light orange nodes indicate genes unique to each set. Functionally related genes grouped under the same Gene Ontology (GO) term are connected by purple edges. (B) Functional enrichment analysis performed in Metascape. Significantly enriched pathways are displayed, with red dots denoting pathways enriched in ADHD PLS1- genes, blue dots for ADHD-C PLS1- genes, and green dots for GWAS-derived ADHD risk genes. All terms are *P*_FDR_ < 0.05. (C) Gene Ontology terms are labeled according to the color scheme of their associated gene set.


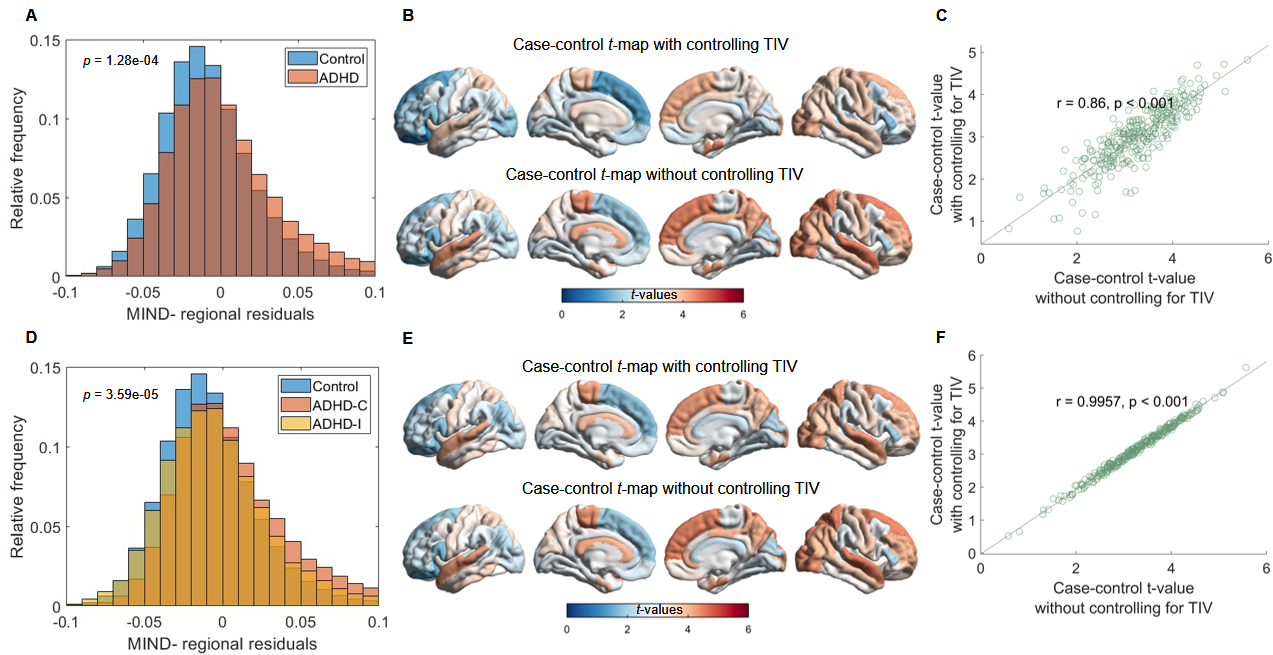


#### **sFig. R7 Validation of the effect of TIV on case–control MIND differences**

(A) Distribution of regional MIND strength (mean MIND per region) without TIV correction in ADHD (red) and TD controls (blue). (B) Regional case–control MIND differences in ADHD after controlling for TIV (top) and without TIV correction (bottom). (C) Spatial correlation (Spearman’s *r*) between regional case–control MIND *t*-statistics with and without TIV correction across 308 brain regions (*r* = 0.86, *p*_spin_ < 0.001). (D) Distribution of regional MIND strength without TIV correction in ADHD (red), ADHD-C (yellow), and TD controls (blue). (E) Regional case–control MIND differences in ADHD-C after controlling for TIV (top) and without TIV correction (bottom). (F) Spatial correlation between regional ADHD-C vs. control *t*-statistics with and without TIV correction (*r* = 0.996, *p*_spin_ < 0.001).


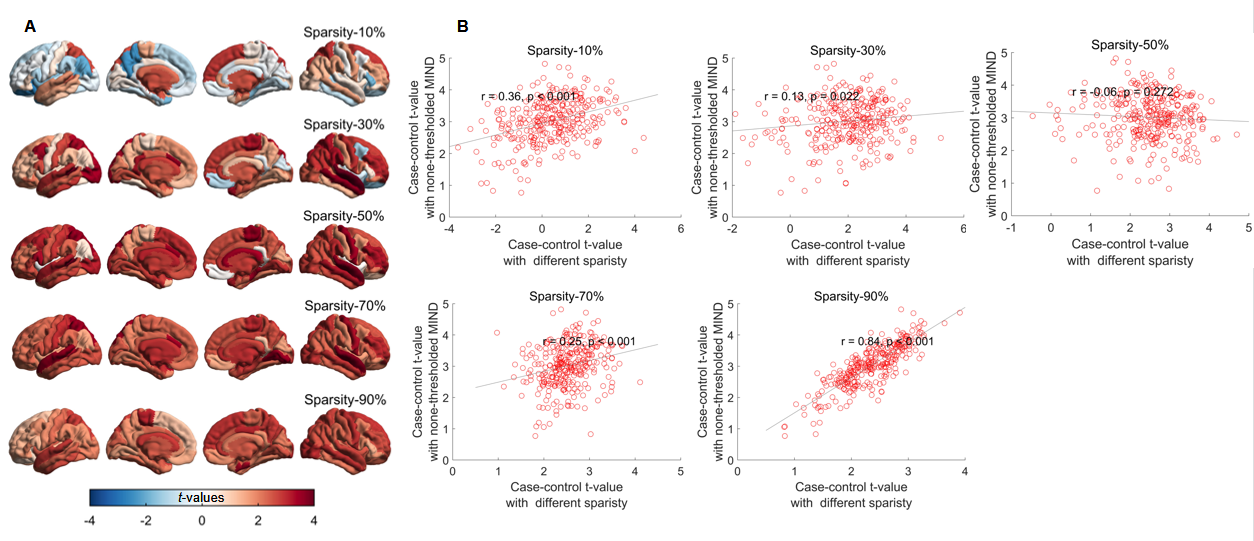


#### **sFig. R8 Replicability of MIND across connection densities in case (ADHD) - control *t* values**

1. Distributions of case (ADHD) - control *t* values across a range of connection densities (from 10% to 90%, in 20% increments). (B) Pearson correlation between case-control *t* values derived from unthresholded matrices and those thresholded at each connection density (10%–90%, in 20% increments).


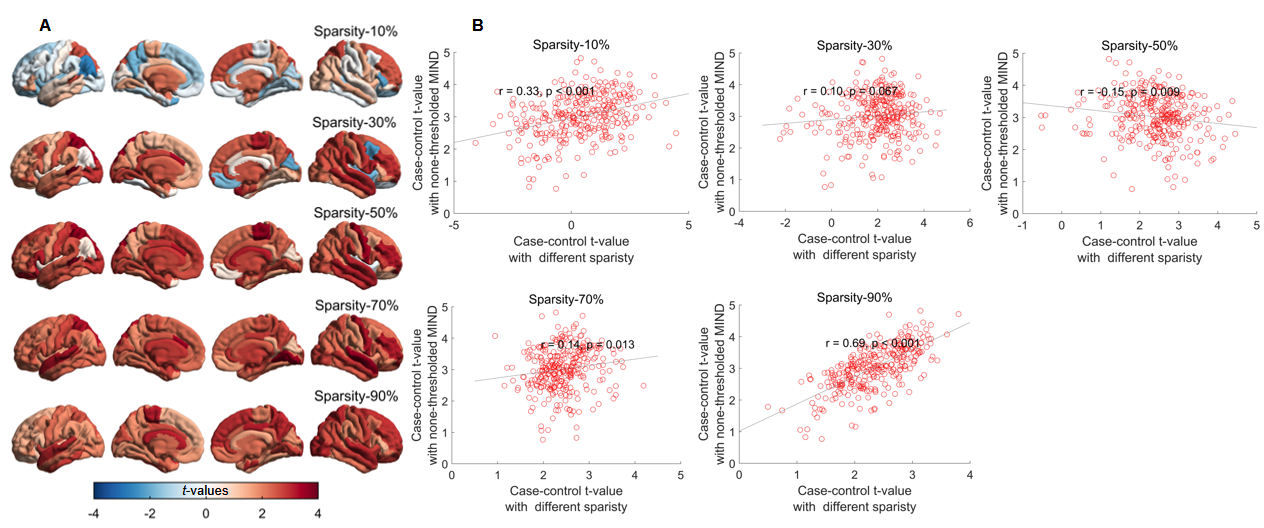


#### **sFig. R9 Replicability of MIND across connection densities in case (ADHD-C) - control *t* values**

1. Distributions of case (ADHD-C) - control *t* values across a range of connection densities (from 10% to 90%, in 20% increments). (B) Pearson correlation between case-control *t* values derived from unthresholded matrices and those thresholded at each connection density (10%–90%, in 20% increments).


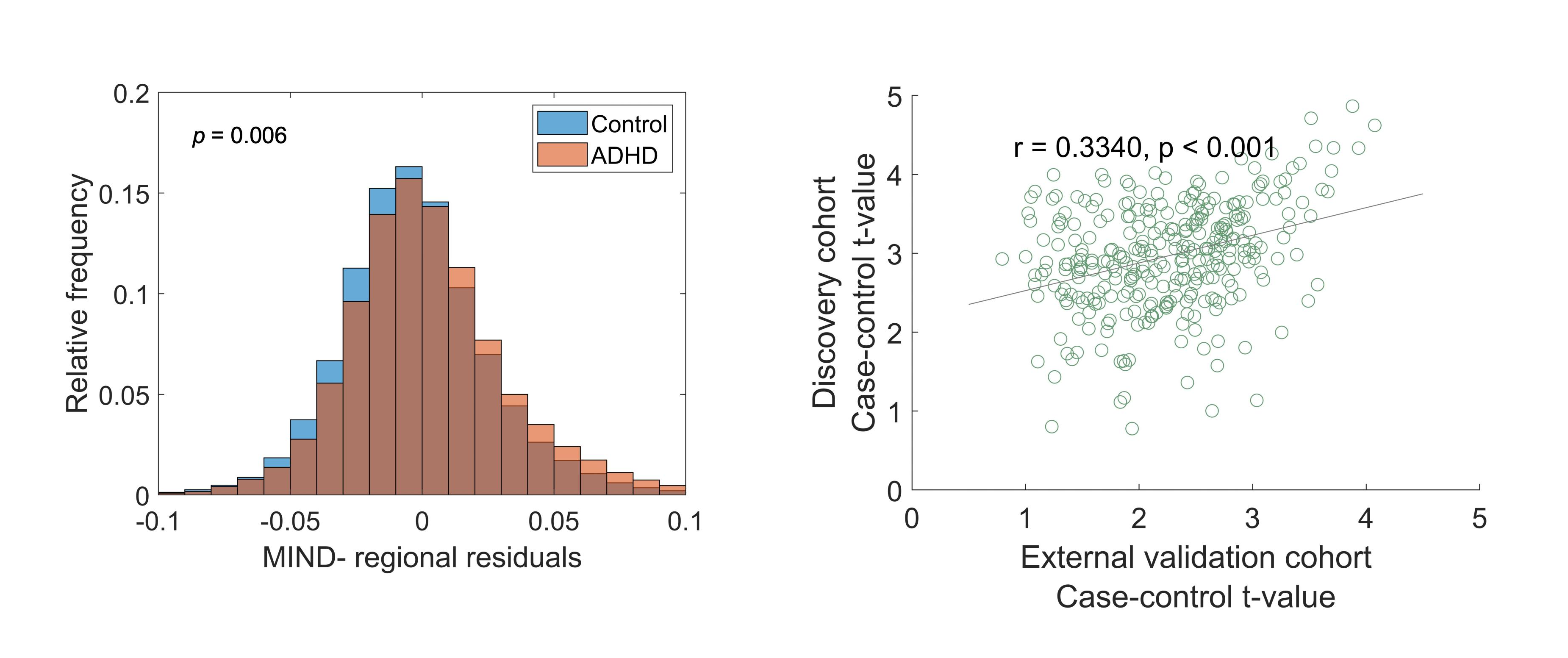


#### **sFig. R10 Reproducibility of ADHD-related MIND alterations in an independent external validation cohort.**

The validation cohort (*N*_total_ = 277, 127 ADHD, 150 TD) was aggregated from four open-access datasets (NeuroIMAGE, ds002424, ds005899, ds004605).

(A) Frequency distributions of regional MIND differences between ADHD and TD (*p* = 0.006). (B) Spearman’s correlation analysis of the case-control *t*-maps of the discovery and validation cohorts (*r* =0.334, *p*_spin_ < 0.001).


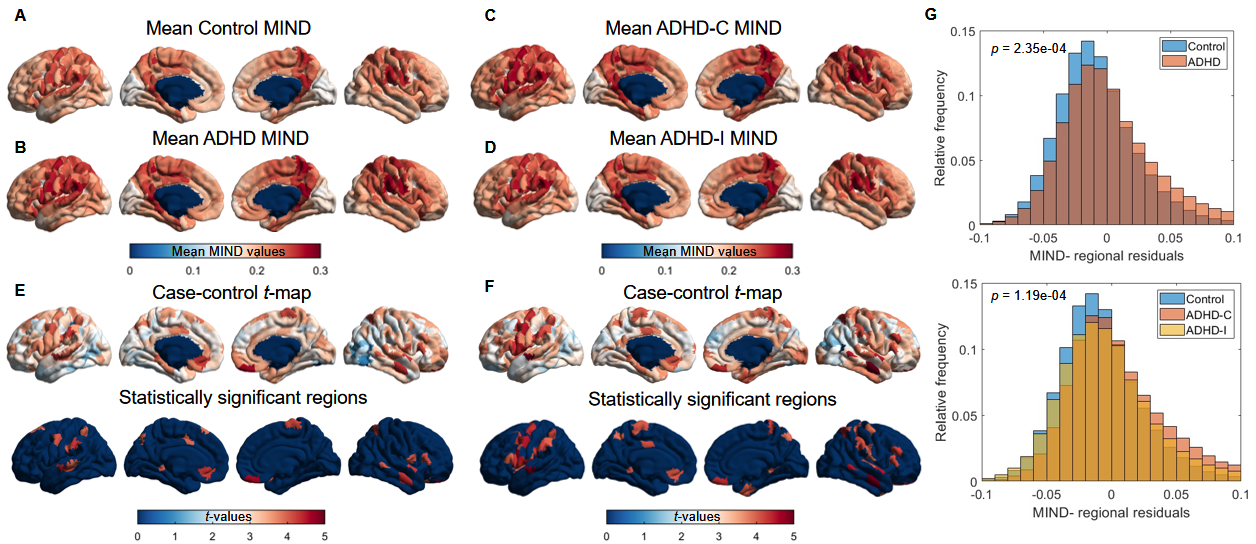


#### **sFig. R11 Schaefer 400 Atlas Reveals Altered MIND Patterns in ADHD and Its Subtypes**

Regional MIND distributions used Schaefer 400 across typically developing (TD) controls (A), the full ADHD cohort (B), ADHD-C (C), and ADHD-I (D) subtypes. (E)Statistical maps of regional MIND differences between the full ADHD cohort and TD controls. Red and blue indicate regions where ADHD > TD and ADHD < TD, respectively (top: unthresholded; bottom: *p*_Bonferroni_ < 0.05). (F) Regional MIND differences between ADHD-C and TD controls, with the same color scheme and thresholding as in (E). (G) Frequency distributions of regional MIND differences between ADHD and TD (top, *p* = 2.35 × 10^-4^), and between ADHD-C and TD (bottom, *p* = 1.19 × 10^-4^), after controlling for age, sex, and TIV.


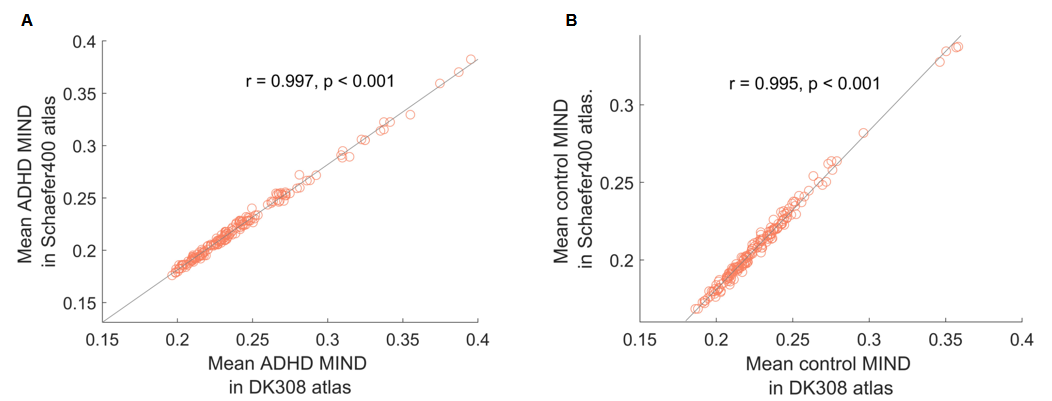


#### **sFig. R12 Cross-Atlas Reliability of MIND Values**

High consistency of mean MIND values between the Schaefer 400 and DK308 atlases in both the ADHD group (A, *r* = 0.997, *p* < 0.001) and the control group (B, *r* = 0.995, *p* < 0.001).
